# Projections of heatwave-attributable mortality under climate change and future population scenarios in China

**DOI:** 10.1016/j.lanwpc.2022.100582

**Published:** 2022-09-05

**Authors:** Huiqi Chen, Liang Zhao, Liangliang Cheng, Yali Zhang, Huibin Wang, Kuiying Gu, Junzhe Bao, Jun Yang, Zhao Liu, Jianbin Huang, Yidan Chen, Xuejie Gao, Ying Xu, Can Wang, Wenjia Cai, Peng Gong, Yong Luo, Wannian Liang, Cunrui Huang

**Affiliations:** a Vanke School of Public Health, Tsinghua University, Beijing, China; b School of Public Health, Sun Yat-sen University, Guangzhou, China; c Shanghai Typhoon Institute, China Meteorological Administration & Shanghai Key Laboratory of Meteorology and Health, Shanghai Meteorological Service, Shanghai, China; d The State Key Laboratory of Numerical Modeling for Atmospheric Sciences and Geophysical Fluid Dynamics (LASG), Institute of Atmospheric Physics, Chinese Academy of Sciences, Beijing, China; e School of Public Health, Zhengzhou University, Zhengzhou, China; f School of Public Health, Guangzhou Medical University, Guangzhou, China; g School of Linkong Economics and Management, Beijing Institute of Economics and Management, Beijing, China; h Department of Earth System Science, Ministry of Education Key Laboratory for Earth System Modeling, Institute for Global Change Studies, Tsinghua University, Beijing, China; i State Key Joint Laboratory of Environment Simulation and Pollution Control (SKLESPC), School of Environment, Tsinghua University, Beijing, China; j College of Earth and Planetary Sciences, University of Chinese Academy of Sciences, Beijing, China; k Climate Change Research Center, Institute of Atmospheric Physics, Chinese Academy of Sciences, Beijing, China; l National Climate Center, China Meteorological Administration, Beijing, China; m Department of Earth Sciences and Geography, University of Hong Kong, Hong Kong Special Administrative Region, China; n Institute of Healthy China, Tsinghua University, Beijing, China

**Keywords:** Climate change, Heatwave, Attributable death, Projection, China

## Abstract

**Background:**

In China, most previous projections of heat-related mortality have been based on modeling studies using global climate models (GCMs), which can help to elucidate the risks of extreme heat events in a changing climate. However, spatiotemporal changes in the health effects of climate change considering specific regional characteristics remain poorly understood. We aimed to use credible climate and population projections to estimate future heatwave-attributable deaths under different emission scenarios and to explore the drivers underlying these patterns of changes.

**Methods:**

We derived climate data from a regional climate model driven by three CMIP5 GCM models and calculated future heatwaves in China under Representative Concentration Pathway (RCP) 2.6, RCP4.5, and RCP8.5. The future gridded population data were based on Shared Socioeconomic Pathway 2 assumption with different fertility rates. By applying climate zone-specific exposure-response functions to mortality during heatwave events, we projected the scale of heatwave-attributable deaths under each RCP scenario. We further analyzed the factors driving changes in heatwave-related deaths and main sources of uncertainty using a decomposition method. We compared differences in death burden under the 1.5°C target, which is closely related to achieving carbon neutrality by mid-century.

**Findings:**

The number of heatwave-related deaths will increase continuously to the mid-century even under RCP2.6 and RCP4.5 scenarios, and will continue increasing throughout the century under RCP8.5. There will be 20,303 deaths caused by heatwaves in 2090 under RCP2.6, 35,025 under RCP4.5, and 72,260 under RCP8.5, with half of all heatwave-related deaths in any scenario concentrated in east and central China. Climate effects are the main driver for the increase in attributable deaths in the near future till 2060, explaining 78% of the total change. Subsequent population decline cannot offset the losses caused by higher incidence of heatwaves and an aging population under RCP8.5. Although health loss under the 1.5°C warming scenario is 1.6-fold higher than the baseline period 1986–2005, limiting the temperature rise to 1.5°C can reduce the annual mortality burden in China by 3,534 deaths in 2090 compared with RCP2.6 scenarios.

**Interpretation:**

With accelerating climate change and population aging, the effects of future heatwaves on human health in China are likely to increase continuously even under a low emission scenario. Significant health benefits are expected if the optimistic 1.5°C goal is achieved, suggesting that carbon neutrality by mid-century is a critical target for China's sustainable development. Policymakers need to tighten climate mitigation policies tailored to local conditions while enhancing climate resilience technically and infrastructurally, especially for vulnerable elderly people.

**Funding:**

National Key R&D Program of China (2018YFA0606200), Wellcome Trust (209734/Z/17/Z), Natural Science Foundation of China (41790471), and Guangdong Major Project of Basic and Applied Basic Research (2020B0301030004).

**Research in context**

**Evidence before this study**

We searched PubMed, Scopus, Web of Science, and Google Scholar from the databases’ inception until June 1, 2022, for articles published in English using the search terms “temperature,” “heat,” “heatwave” AND “mortality,” “death,” “attributable death,” “attributable risk” AND “projection,” “estimation,” and “prediction.” Many studies have consistently projected that human mortality caused by climate conditions exceeding the offensive heat threshold will be significantly aggravated under climate change, while mitigation-related emission scenarios will largely determine the extent of the future mortality burden. In addition, previous studies have demonstrated that changes in heat-related mortality are driven by climate warming. Because most previous studies in this field were conducted in developed countries such as the United States and Europe, the evidence might not apply directly to China, which is a populous country with a rapidly evolving socioeconomic context, an aging population, and climatic diversity. Several previous studies have projected heat-related mortality in many cities in China under moderate and high emission scenarios using global climate models. However, little is known about the spatiotemporal dynamics of the national mortality burden with regional climate data and credible population scenarios that consider fertility-encouraging policies. In addition, the factors driving changes in heatwave-related deaths in China remain unclear in the long term, impeding current understanding of severe heatwaves.

**Added value of this study**

The current study provides a comprehensive assessment of the future mortality burden of heatwaves across China under different climate change scenarios, on the basis of more credible regional climate and population change data than global climate models and a constant population scenario. We observed pronounced increases in heatwave-related mortality across the country over time even under a low emission scenario, particularly in the coming years. However, following the stringent 1.5°C warming pathway could reduce quantities of deaths compared with all selected RCP emission scenarios. In addition, to fully understand the contributors of future heatwave-attributable deaths, we further showed that the changes in attributable deaths are primarily driven by climate effects and population aging. Integration of these elements provides plausible nationwide estimates of heat-related health risks for future generations in China. Our findings add the ambitious warming targets to previous RCP-based assessments of heat-related health insults in China.

**Implications of all the available evidence**

This study contributes to the ongoing debate from the Paris Agreement about the need to urgently curb climate change by mitigation and minimize its health consequences. As global warming and population aging speed up, the health loss from dangerous heatwaves in China is likely to see a continual rise even under a low emission scenario. Mitigation and adaptation policies can be targeted toward locations with disproportionate risk, or where risk is expected to increase, and vulnerable older populations to minimize the effects of extreme heat events on human lives. Notably, limiting global warming to 1.5°C without overshoot could bring substantial health benefits, suggesting that achieving carbon neutrality by mid-century is an important target for sustainable development in China.

Alt-text: Unlabelled box

## Introduction

Climate change poses widespread and frequently aggressive threats to public health, and one primary impact is the health burden of high temperatures on mortality.^1^ Extreme heat events, widely known as heatwaves, are projected to increase dramatically in frequency, severity and duration under climate warming, causing more pervasive health and socioeconomic consequences in the future.^1^, ^2^, ^3^ A large number of studies have projected significantly exacerbated mortality effects caused by increasing heatwaves in varied settings, particularly in subtropical developing countries under high emission scenarios.^4^^,^^5^

China is a vast country containing wildly differing climatic regions and topographical extremes. During the past century, China has seen a rapid increase in surface air temperature with large decadal variability; heat extremes have become more likely and more intense, mainly owing to climate change related to atmospheric circulation anomalies and human activities, as well as local factors such as urbanization.^6^^,^^7^ As the largest developing country, China also faces drastic socioeconomic changes in population aging and migration, which will jointly aggravate these health impacts.^8^ To combat climate change and promote sustainable development, China is scaling up its nationally-determined contributions, aiming to peak carbon emissions by 2030 and reach carbon neutrality by 2060.^9^ This demands the implementation of an aggressive 1.5°C restrict pathway or a feasible 2°C alternative pathway to meet the targets of the Paris Agreement and avoid the climate crisis.^10^

Previous studies have projected heat-related mortality in many cities in China, indicating growing risks connected to extreme heat under future conditions of continued warming.^11^, ^12^, ^13^, ^14^, ^15^ However, aggregated results from multiple cities cannot reflect the death burden across the whole country, and do not apply to large areas with diverse climates and demographic patterns.^11^^,^^13^^,^^14^ In addition, many previous studies have used coarse global climate models (GCMs) with unsatisfactory regional climate performance, and directly adopted population scenarios from the global projection database without considering birth policy shifts, resulting in biased results that are not in accord with China's national circumstances.^11^^,^^13^ In addition, it is insufficient to simply enumerate the death burden in a certain future period, and analyzing main driving factors triggering the changes is more relevant for enabling policymakers to take action.

To fill this gap in current knowledge, we projected future heatwave-attributable mortality in China based on regional climate models (RCMs), considering China's future climate mitigation policies and demographic structure changes. Specifically, we aimed to reveal the spatiotemporal dynamics of future heatwave-related mortality under different climate change scenarios, and to explore the drivers behind the identified pattern of changes. To produce more policy-relevant findings, we further examined the health benefits on mortality of achieving the 1.5°C goal. This study belongs to an initiative of the Lancet Countdown Asia Centre, which aims to provide a multi-dimensional projection of the future health risks from climate change in China.

## Methods

In this modeling study, the latest 0.5-degree gridded climate data were derived from RCMs under three Representative Concentration Pathways (RCP) scenarios, while the population data employed the latest birth rate trends after the implementation of the three-child policy. To better comprehend the projected health impacts for the future, we analyzed the sources of uncertainty and drivers of changes in deaths ascribed to heatwaves. We then compared the differences in heatwave-attributable deaths under a 1.5°C warming scenario with three RCP scenarios to quantify the benefits of strict future mitigation policies.

### Data collection

#### Study area

The study area includes 3,829 grids of 0.5-degree resolution across 31 provinces in mainland China. The whole country is divided into seven climate zones, including humid monsoon climate with cold winters, semi-arid monsoon climate, north subtropical monsoon climate, mid subtropical monsoon climate, south subtropical monsoon climate, arid and semi-arid continental climate, and plateau climate.^16^

#### Mortality records

The daily mortality data in all 31 provincial capitals from 2007 to 2013 were collected from the Chinese National Center for Disease Control and Prevention. The underlying cause of death was coded on the basis of the 10th Revision of the International Statistical Classification of Diseases and Related Health Problems (ICD-10). The daily death number was further categorized by gender and age group. Details of mortality data can be found in a previous study by Yang et al.^17^

#### Projected daily temperature series under climate change scenarios

The daily temperature data at 25 km are derived from a regional climate model (RegCM4.4) over the CORDEX-East Asia domain driven by three CMIP5 GCMs, including HadGEM2-ES, MPI-ESM-MR, and NorESM1-M.^18^ Using regional climate models not only produces climate variables at spatially high-resolution grids, but shows better regional climate performance over China (Appendix).^18^ The climate simulations cover the period 1986–2005 (historical simulation) and 2006–2100 (future scenario simulations) under RCP2.6, RCP4.5, and RCP8.5 to represent low, moderate and high greenhouse gas (GHG) emission scenarios, respectively.^19^ For the 1.5°C climate goal related to achieving carbon neutrality by mid-century, the temperature in 2021–2040 under the RCP2.6 scenario is used to represent the climate if global warming reaches 1.5°C compared with pre-industrial levels (Appendix).^20^

The model outputs were bias-corrected and downscaled through bi-linear interpolation at 0.5° × 0.5° resolution and linear interpolated by day of the year,^21^ which led to an improvement of RCMs in reproducing the observed spatial pattern and long-term average of climatic variables (Appendix Method, Figure S1). The observed temperature data were from the latest global atmospheric reanalysis version 5 of the European Centre for Medium-Range Weather Forecasts.^22^

#### Population projections in China

The assumption of Shared Socioeconomic Pathway 2 was adopted for the medium population scenario because it depicts a very likely development scenario for future China.^23^^,^^24^ In addition, the high and low population scenarios were derived by assuming high and low fertility rates to reflect the impact of the latest birth policies implemented in China.^23^ We then derived the prospects of 1 km resolution during 2010–2100 and further aggregated the results to 0.5° × 0.5°. We also collected historical population data during 1986–2009 from the global hybrid gridded demographic datasets.^25^

Data on the demographic characteristics (e.g., yearly mortality rate) in each province during 1986–2019 was extracted from the Chinese statistical yearbooks,^26^ which was further matched with a 0.5-degree grid. We assumed that the future mortality rate is the same as the average value of the 2010s because the value has been stable over the past decade.

### Data analysis

#### Heatwave definition

Heatwave was defined as a consecutive period of at least 3 days during which the daily maximum temperature exceeded the 92.5^th^ percentile of the reference period (1986–2005). This definition is optimal for its capacity to capture the real pattern of health effects at a national scale derived from the best model fit of exposure-response relationship (Appendix).^17^ Because almost all heatwaves occur in warm seasons,^27^ we limited our research to May to September.

#### Historic heatwave-mortality relationships

Relative risk (RR) represents the increase in the risk of mortality resulting from heatwave days compared with non-heatwave days. We analyzed location-specific exposure-response functions between heatwave and mortality using a Poisson generalized linear model, and adjusted for confounders such as air pollution index and relative humidity (Appendix). Parameter estimates for 31 provincial capitals were then provided to represent corresponding provinces.

Next, pooled effects of heatwave on mortality at 31 provinces across seven climate zones were produced using a meta-analysis to identify regional patterns of mortality risk in response to heatwaves. When the Q test yielded statistically significant results and I^2^ ≥ 50%, the random effects model was applied. Otherwise, the fixed effects model was used (Appendix Method, Figure S2). Thus, we obtained gridded RR by matching climate division-specific RR with the grid in different climate zones.

#### Projections of future heatwave-related mortality

Using the daily maximum temperature output from each RCM for the future, we calculated the number of heatwave days per year from 1986 to 2100. We calculated expected deaths during heatwave days per year for each set of climate scenarios (three RCPs and 1.5°C pathway) and RCMs, from which we can compute the statistics of change. The number of attributable deaths (AN) during given heatwave periods was calculated for each grid every year as follows:

$$AN_{y,p}=\sum^{m}Pop_{y,p}\times Mort_{d,m,y,p}\times HW_{m,y,p}\times AF_{y,p}$$

where *Pop_y,p_* refers to the grid cell-level population size in year *y* and in grid *p. Mort*_y,_*_p_* is the yearly baseline mortality rate, which is multiplied by the monthly mortality proportion then divided by days in month *m* to obtain the daily mortality rate *Mort_d,m,y,p_* in month *m* of year *y. m* ranges from May to September. We assumed the mortality rate to be unchanged from 2020 until the end of the century. *HW_m,y,p_* is the heatwave days in month *m* of year *y* in grid *p. AF_y,p_* is the attributable fraction of heatwave, which is calculated as:

$$AF_{y,p}=\left(RR_{y,p}-1\right)/RR_{y,p}$$

where *RR_y,p_* refers to RR in year *y* and in grid *p*.

#### Temporal and spatial analysis

We used Monte Carlo simulations to produce 1,000 samples of coefficients on the heatwave-mortality association, assuming that the estimated model coefficients followed a normal distribution, then generated results for each climate model and each year from 1986 to 2100. We reported the results as point estimates, using the average of RCM results driven by three CMIP5 GCMs on the basis of decades, RCP, and population scenarios. The 95% empirical confidence intervals (CIs) were defined as the 2.5^th^ and 97.5^th^ percentiles of the empirical distribution across coefficient samples and climate models.

Spatial analysis was then performed for grid cells. For individual combinations of RCPs and population scenarios, the 20-year averaged mortality burden was estimated for the baseline period (1986–2005), the early (2021–2040, centered on 2030), middle (2051–2070, centered on 2060), and late future (2081–2100, centered on 2090). Subsequently, we calculated the percentage change for the future years relative to the baseline period. Finally, we conducted a spatiotemporal comparison on the basis of different climate scenarios.

#### Decomposing the drivers of heatwave-attributable deaths

We dissected the contributions of drivers including climate effects, population size, and aging, to the change in attributable deaths using the decomposition method.^28^ This approach estimated the contribution of different factors by sequentially introducing each factor into the AN equation. The difference between each consecutive step provided an estimate of the relative contribution of each factor. We then estimated the results under all sequence permutations of the three factors. The final estimation of contributions from different factors was the average of the results for all sequences (Appendix).

#### Uncertainty assessment

Estimation of the mortality burden under climate change involves numerous uncertainties coming from the complex interactions among population change scenarios, climate models and scenarios, and uncertainty in estimated future heatwave-mortality associations. We considered three population scenarios in the future with various fertility rates. Other sources of uncertainty in attributable deaths are related to the estimates of RR parameters regarding CIs, and the variability in temperature projections between models in specific climate scenarios.^5^ We assumed no change in baseline mortality rate or adaptation to extreme heat. The source of uncertainty at different time points was then analyzed using a method similar to the decomposition of driving factors.

### Role of funding source

None.

## Results

**Figure 1:**
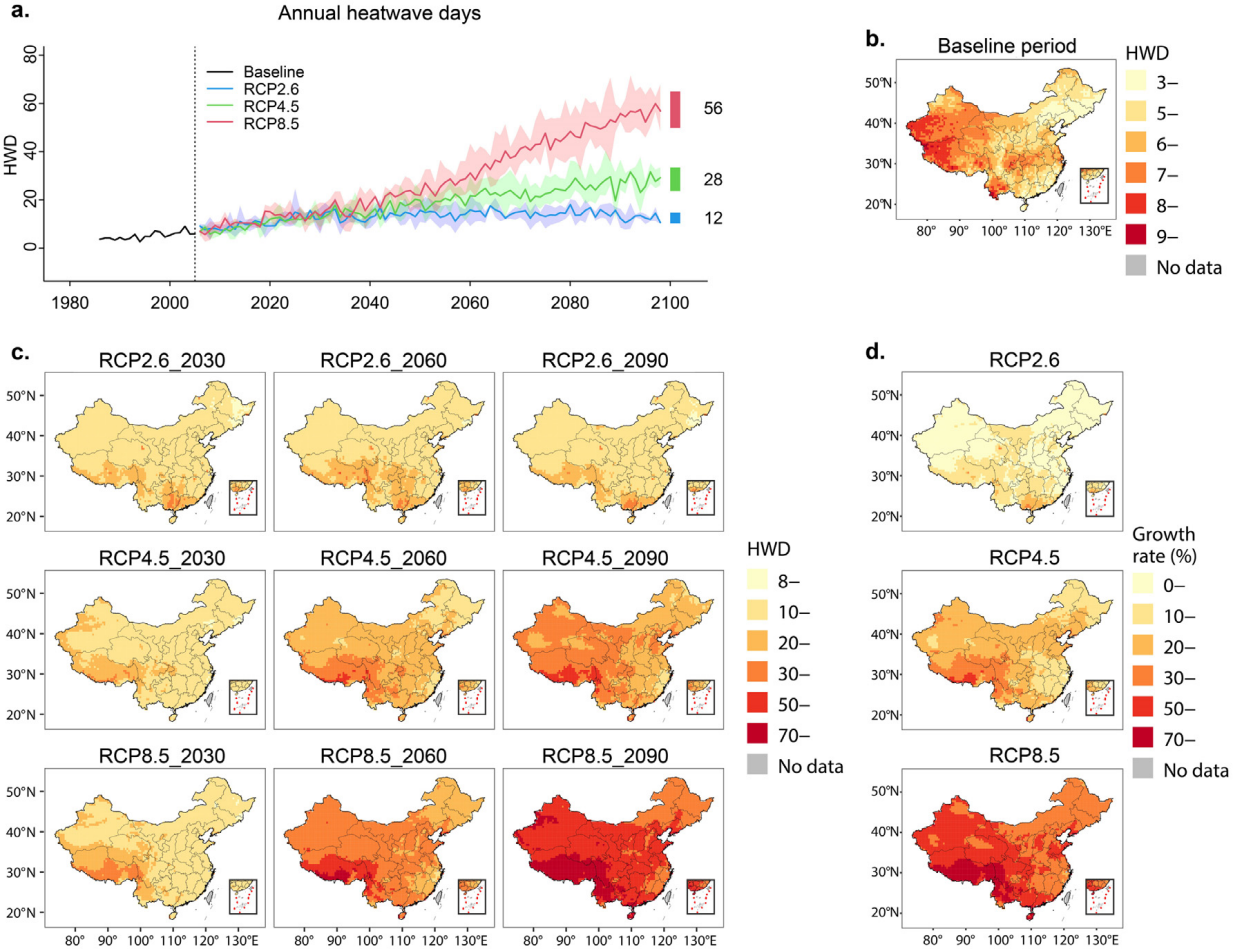
Projections of yearly heatwave frequency under different climate change scenarios in China. **a**, Temporal trends of national annual heatwave days under three RCP scenarios from 1986 to 2100. Solid lines denote the estimated mean annual heatwave frequency across the three RCM-specific modeled series. The shaded area shows variability, corresponding to the range for each year. The solid black line corresponds to the trend on the baseline period (1986–2005). The vertical colored lines on the right correspond to the average annual maximum and minimum for 2090–2099 under each scenario. **b**, Spatial differentiation of multi-years-average heatwave days in 0.5-degrees grid during the baseline period. **c**, Spatial differentiation of multi-years-average heatwave days in grid in 2030 (2021–2040), 2060 (2051–2070), 2090 (2081–2100). **d**, Changes in heatwave days in 2090 (2081–2100) relative to the baseline period. Growth rate (%) = (HWD_2090_- HWD_baseline_) / HWD_baseline_ × 100%. HWD, heatwave days; RCP, representative concentration perspectives.

Overall, heatwave frequency showed a similar fluctuating upward trend until 2030 under three RCP scenarios. Subsequently, the number of heatwave days under a high emission scenario (RCP8.5) was consistently higher than that under a moderate emission scenario (RCP4.5) and the green road with significant carbon emission reduction (RCP2.6). Under RCP8.5 and RCP4.5, the annual frequency of heatwave occurrence increased 10.3 and 5.2 times in 2090 compared with the 1986–2005 baseline period, respectively. Under RCP2.6, the annual frequency of 14 heatwave days only increased 2.6-fold (Figure 1a).

Nationally, 16.2% of the country experienced more than 10 heatwave days (multi-year average) during the baseline period, which was projected to increase to 85.7%, 99.7%, and 100% in 2090 under RCP2.6, RCP4.5, and RCP8.5, respectively. All regions exhibit persistent increasing trends, with the strongest increase in south and southwest areas and larger areal coverage to the east and central regions (Figure 1c & d). Moreover, some areas with large populations and developed economies, such as east and central China, experienced the highest population exposure (Figure S3c).

**Figure 2:**
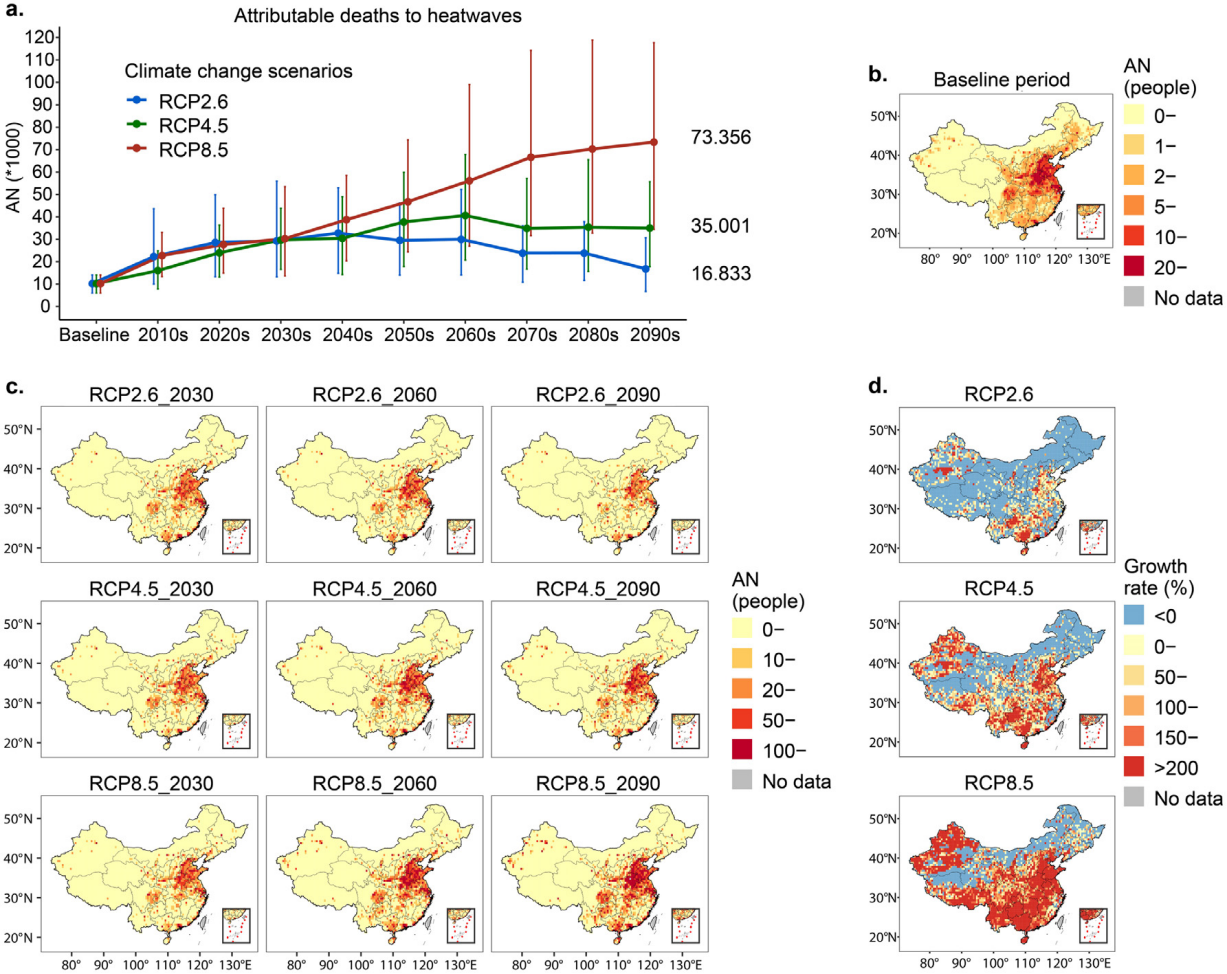
Projections of decade-average deaths attributable to heatwaves in China. **a**, Temporal trends of national decade-average heatwave-attributed deaths under three RCP scenarios from 1986 to 2100 with middle-fertility assumption. Solid broken lines denote the estimated mean decade-average attributed deaths across the three climate models. The solid vertical line represents the 95% empirical confidence interval (CI) of the ensemble of models. Baseline refers to the baseline period (1986–2005). The number marked on the right is the average attributable deaths from 2090 to 2099. Monte Carlo simulations generating 1,000 samples were computed to produce the empirical CI. **b**, Spatial differentiation of multi-years-average attributed deaths in 0.5-degrees grid during the baseline period. **c**, Spatial differentiation of multi-years-average attributed deaths in grid in 2030 (2021–2040), 2060 (2051–2070), 2090 (2081–2100). **d**, Changes in attributable deaths to heatwaves in 2090 (2081–2100) relative to the baseline period. Growth rate (%) = (AN_2090_- AN_baseline_) / AN_baseline_ × 100%. RCP, representative concentration perspectives; AN, number of deaths attributable to heatwaves.

Unlike the general increase in heatwave frequency, the resulting pattern of total heatwave-attributable deaths differed between emission scenarios (Figure 2a). Under RCP8.5, the death toll will continue to rise, whereas under RCP2.6 and RCP4.5, it will increase until 2040–2060, then decrease. Importantly, even under RCP2.6, the burden will increase to 31,278 deaths by mid-century, with a growth rate of 205% compared with the baseline period. By the end of the century, mortality from heatwaves is projected to increase by 6.7, 3.2, and 1.9 times, under RCP8.5, RCP4.5 and RCP2.6, respectively, with the corresponding number of annual deaths jumping from 10,264 (5,996–14,094) in the baseline period to 72,259 (33,911–119,394), 35,025 (16,834–59,865), and 20,303 (9,153–34,179) by 2090 (Table S1). The temporal trend in percentage change is similar to that in attributable numbers, with values of 606%, 242%, and 98% in 2090 under RCP8.5, RCP4.5, and RCP2.6, compared with the baseline period (Table S2, Figure S4).

The mortality burden exhibited a strong spatial heterogeneity, with half (47.4%–51.7%) of all deaths concentrated in east and central China under any scenario, covering China's developed urban agglomerations (Shandong Peninsula, North China Plains, Yangtze River Delta). Among the 31 provinces, Henan and Shandong consistently suffer from the most severe health burden over time, accounting for 14.4% and 11.3% of the national total respectively (Figure 2c). Except for northeast areas, almost every province in China will see an increase in attributable deaths, but the growth is higher in south and southwest (Figure 2d). Compared with the baseline period of 1986–2005, the rate of increase in some provinces (e.g., Hainan, Guizhou, and Guangxi) will be tripled in 2090 (Figure 2d, Figure S5).

**Figure 3:**
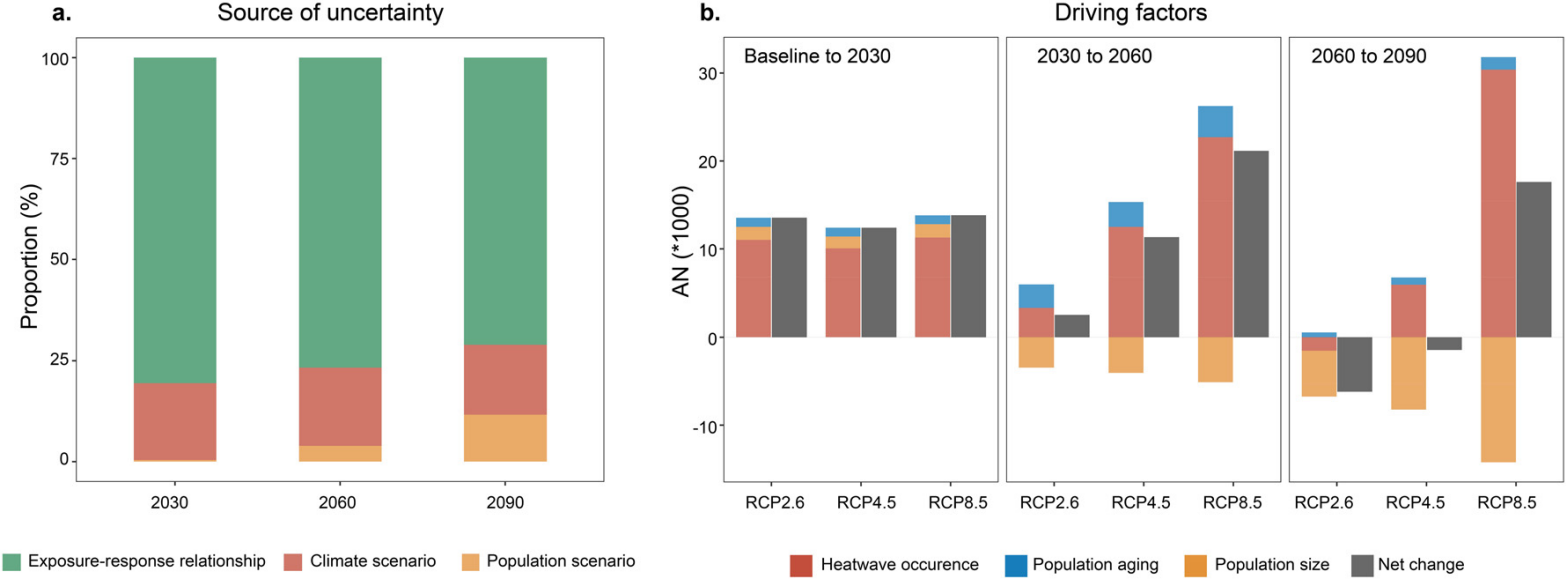
Uncertainty sources and factors driving changes in heatwave-attributable deaths. **a**, The sources of uncertainty in attributable deaths in 2030, 2060, and 2090. **b**, Multi-years-average changes in heatwave-attributed deaths, independently driven by future climate, population size and aging changes during historical time (1986–2005) to 2090 under three climate change scenarios. AN, number of deaths attributable to heatwaves; RCP, representative concentration perspectives.

The changes in attributable deaths are jointly driven by climate warming, population size, and aging, with climate effects playing a major role (Figure 3b). In the near future, the increase in attributable deaths will be primarily caused by the higher incidence of heatwaves under future warming conditions. Climate effects explained 81.5% of the increase in attributable deaths in the historical period to 2030, and 74.7% of the increase in the period from 2030 to 2060. This slight decline is caused by more severe aging as well as the population peaks in the 2030s, and plays an opposite role thereafter. In the far future of 2060–2090, climate effects still exert a paramount influence, although the situation becomes more complex. The decline of attributable deaths during this period in RCP2.6 is caused by the direct effects of reduced heatwave frequency and total population. Under RCP4.5, death numbers remain relatively steady as the climate and population effects maintain a balance. Under RCP8.5, the decline in population size cannot offset the losses caused by elevated heatwave frequency (explaining 95.6% of the growth in attributable deaths) and an aging population (explaining 4.4% of the growth in attributable deaths). As the effects of climate play a major and profound role in future health burden, China is at a critical crossroads in deciding which pathway to follow.

Intriguingly, we found considerable variability in the projections of annual heatwave mortality, which enhance gradually over time with point estimates ranging from 25,000 to 86,000 deaths per year in 2090. In addition, the sources of uncertainty between different RCP scenarios are similar (Table S3). In particular, the largest source of variation appeared to be the statistical noise from exposure-response parameters, followed by variation caused by different climate model implementations and the choice of population scenarios, which accounted for 76.7%, 18.1%, and 5.3%, of variation, respectively (Figure 3a). In subgroup populations, the general temporal trend is similar to that in the total population under three RCP scenarios (Figure S6). Notably, there is one exception for elderly people aged over 75 in the distant future under RCP4.5. The death toll in this population will not decline, but will continuously increase until the end of the century, to 9,630 in 2060 and 10,128 in 2090, which highlights the necessity of paying more attention to the growing aging population.

**Figure 4:**
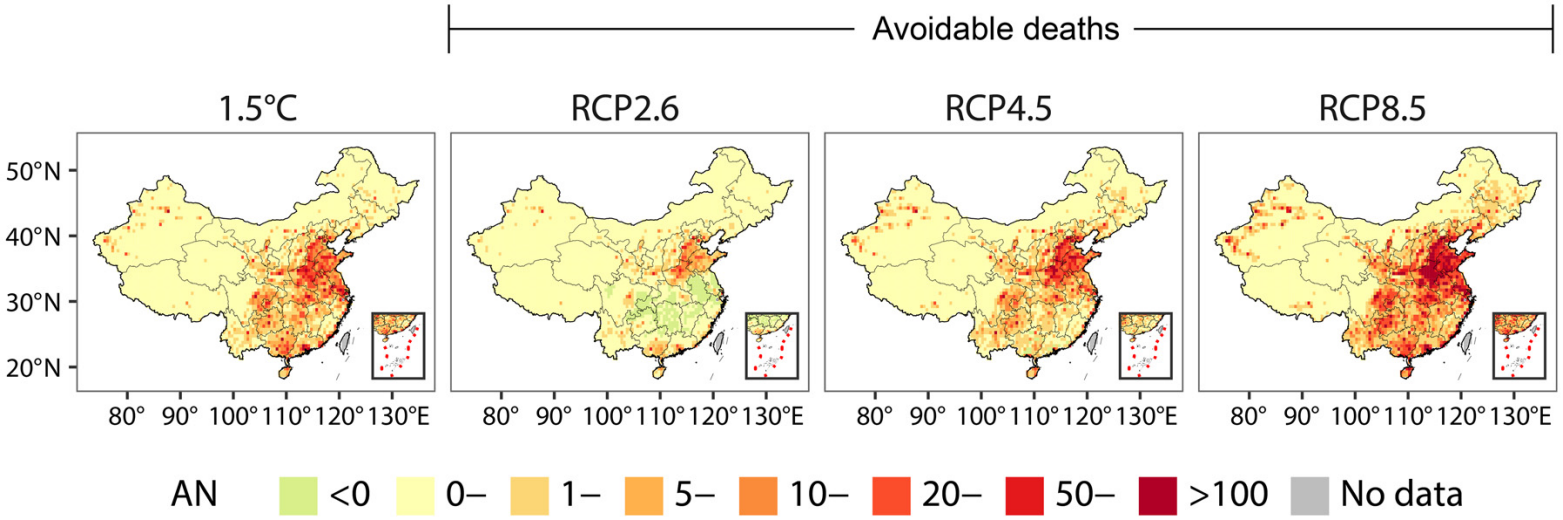
Projections of heatwave-attributable deaths at the global warming levels of 1.5°C and three RCP scenarios. **a**, Spatial differentiation of multi-years-average attributed deaths in 0.5-degrees grid under 1.5°C scenario in 2090. **b**, Distribution of avoidable deaths related to heatwave under 1.5°C scenario compared with RCP2.6, RCP4.5 and RCP8.5 in 2090. AN, number of deaths attributable to heatwaves; RCP, representative concentration perspectives.

We further estimated the death burden under a more sustainable development pathway at the 1.5°C warming level, revealing that the estimated 16,769 deaths in 2090 is still 1.6-fold higher compared with the baseline period. However, limiting the temperature rise to 1.5°C can reduce the annual health losses in China by 3,534, 18,257, and 55,492 deaths compared with RCP2.6, RCP4.5, and RCP8.5, respectively (Figure 4). Spatially, north, east, and central China are the regions that will benefit the most if a stringent 1.5°C warming target is met, with avoidable deaths accounting for 63%–68% of those nationwide (Table S4).

## Discussion

To the best of our knowledge, this is the first study to holistically assess the health impacts of heatwaves across China under different climate change scenarios, including the most ambitious climate goal, on the basis of credible regional climate and population projection data. The results of our model indicated that heatwaves led to a rise in mortality burden even in low or moderate emission scenarios, especially for the next several decades until mid-century. However, a substantial number of deaths can be avoided under the 1.5°C warming scenario by achieving carbon neutrality in the middle of this century. Climate effects and risk estimates are the most influential factors for the changes and ranges in mortality burden, respectively.

The number of heatwave-attributable deaths in China will dramatically increase, even under a low emission scenario, whereas thousands of deaths could be avoided by achieving the 1.5°C target. A multi-region analysis showed that limiting warming below 1.5°C could reduce the heat-related mortality impacts by 0.1%–2.1% compared with 2°C.^29^ In China, a previous projection study showed that the additional warming from 1.5°C to 2°C will lead to more than 27,900 annual extra heat-related deaths. This number is higher than our estimates, possibly because we used a more definite extreme heat definition rather non-optimal temperature.^13^ Because China is devoted to achieving carbon-neutrality in 2060 to meet the 1.5°C target, future climate change may differ from traditional scenarios, and relevant evidence is urgently needed to support these climate mitigation efforts.

We found that east and central China are at a higher risk for mortality burden, with the colder regions of north China receiving substantial benefit from achieving the 1.5°C goal. Likewise, a national study in the United States suggested that cities located in cold northern areas, such as Boston, Chicago and Detroit, could see a significant fraction of avoided heat-related deaths if the current warming trajectory of 3°C could be reduced to 1.5°C or 2°C, in accord with the Paris Agreement thresholds.^30^ In China, previous health studies regarding the 1.5°C goal have only reported the scale of heatwave-attributable deaths in major cities, rather than considering spatial variation across the country to provide local health risk information.^13^ The current results highlight the need for the international community to cooperate to control global warming, and for individuals to adjust their lifestyles and embrace low-carbon living to actively strive to achieve the 1.5°C target.

There is a general trend for an increase in premature deaths under different climate scenarios. Thus, it is important to explore the main drivers of this change. Previous studies have mainly focused on the drivers of the attributable burden in the fields of air pollution or natural disasters. For example, studies of air pollution-related premature deaths revealed that pollution emissions were the primary drivers in China between 2002 and 2017, followed by population aging.^31^ In addition, heatwave-attributable fatalities in Europe are projected to exhibit a sharp rise, with global warming and population growth accounting for 90% and 10% of the increase, respectively.^32^ Similarly, the current findings indicated that climate effects were the main driver for changes in mortality from heatwaves. Therefore, a considerable number of deaths could be avoided by climate mitigation through emission reduction, and early action on emissions will improve future options. Moreover, even if strict mitigation policies are adopted immediately, there will still be a continued increase in attributable death burden in the coming years because of accumulation of past GHG emissions and a growing elderly population. Therefore, the vulnerable population needs to be given more attention, and adaptation strategies should be developed for the negative impact of population aging accompanied by the increasing prevalence of chronic diseases throughout the century.^33^

Evaluating and managing uncertainty is a major challenge for projecting future heat-related mortality.^34^ Previous studies have used variance decomposition methods to obtain reasonable ranges for heatwave effects on health. For example, Wu et al. (2014) attributed 32%, 24%, and 22% of the uncertainty to heatwave-mortality association parameters, emission scenarios and heatwave definitions in the eastern United States.^35^ In addition, another analysis qualitatively reported that the choice of emission scenarios and future population are the main sources of uncertainty in mortality projections in Korea.^36^ The sources of uncertainty are currently unclear, and the current study provides quantitative evidence from China suggesting that parameters and models are the main sources of uncertainty. Because the uncertainty in models can be reduced to a certain extent by multi-model ensemble averaging, it will be important to improve the accuracy of these parameters in the future.

Several limitations in the current research should be noted, because the assumptions in the study design might be violated in the future. First, we assumed that the baseline rate of mortality on heatwave days will be the same in the future as it was for the 2010s. Because China faces severe aging in the future along with advanced medical care, the resulting attributable deaths may have been underestimated or overestimated. Second, we assumed that there will be no adaptation to extreme heat, because epidemiological evidence regarding the extent to which short- or long-term acclimatization mechanisms alter the population risk of heatwaves is limited and inconsistent.^37^^,^^38^ This assumption may have led to overestimation of heatwave impacts for the future, if air-conditioning becomes much more prevalent in China. Third, we assumed that the mortality burden attributable to heatwaves will not be affected by other environmental factors such as air pollution. Because there is an interaction effect between heatwaves and air pollution, the results could be biased, depending on future pollution control policies. Nevertheless, assuming unchanged relative risks and mortality rates and no combination effects with air pollution may better reflect the impacts of climate change in a way that is relevant to policy making.

Regarding future research prospects, studies of historical associations and even future projections indicated that increased health risks were associated with heat events such as compound hot extremes, especially in the context of anthropogenic emissions and urbanization.^39^^,^^40^ However, heatwave-related mortality is the most direct and explicit health impact of climate change. The prediction of China's urbanization progress remains relatively unreliable because of the uncertainty in future climate and population policies along with the complexity in the impact of emergencies like infectious disease pandemics. In the future, more studies will be needed to provide a policy-relevant reference for increasing metropolises when regional urbanization data become available.

In conclusion, climate change will continuously harm human health over the next century, particularly in China's rapidly aging society. Even under a strict low emission scenario, the attributable death burden will increase in the coming decades because of accumulation of past GHG emissions and a large elderly population. However, following the stringent 1.5°C warming pathway could minimize the death toll, especially in northern regions of China. Therefore, policymakers need to tighten climate mitigation policies tailored to local conditions and prioritize pathways toward carbon neutrality and sustainable development. Meanwhile, the findings of this study provide insights for government and communities in designing heat-risk management and interventions, and strengthening technical and infrastructural adaptation measures, especially for elderly people.

## Contributors

CH and WC initiated the study, and contributed to methodological design and coordination. HC, LZ, LC, JB, JY, JH, YL, XG, YX, YC, and CW collected the data. HC, LZ, LC, HW, KG, and YZ cleaned, calibrated and verified the data. HC performed the statistical analysis and drafted the paper. CH, WC, and ZL contributed to interpretation of results and editing the paper. CH, PG, and YL supervised the study. All authors have read the final version of the manuscript and approved the submission.

## Data sharing statement

The dataset generated and analyzed during this study is available from the corresponding authors upon reasonable request. The mortality data can be obtained from China CDC under the agreement to not engage in the unauthorized distribution of the raw data to a third party and to use the data for scientific research only (http://www.phsciencedata.cn/Share/en/index.jsp). Demographic data can be accessed from Chinese national and provincial statistical yearbooks or population census. Climate and population data in the history and future, and the code are available on request from the corresponding author.

## Declaration of interests

The authors declare no competing interests.

## Appendix Supplementary materials

Image, application 1

Image, application 2

Image, application 3
