# Supplementary material for: Projections of heatwave-attributable mortality under climate change and future population scenarios in China
Source: Lancet Reg Health West Pac. 2022 Sep 5;28:100582. doi: 10.1016/j.lanwpc.2022.100582 (PMC9465423; doi:10.1016/j.lanwpc.2022.100582)
Supplement: Supplementary file 1 [file mmc1.docx]

**Supplementary Data- Captions:**

1. Supplementary methods & results
2. Chinese abstract
