# Supplementary material for: Projections of heatwave-attributable mortality under climate change and future population scenarios in China
Source: Lancet Reg Health West Pac. 2022 Sep 5;28:100582. doi: 10.1016/j.lanwpc.2022.100582 (PMC9465423; doi:10.1016/j.lanwpc.2022.100582)
Supplement: Supplementary file 2 [file mmc2.docx]

**Supplementary Information**

# 1. Supplementary Methods

## 1.1 Summary of analytical steps performed in the study.

We projected death burden attributable to heatwaves under Representative Concentration Pathway (RCP) 2.6, RCP4.5, RCP8.5 and 1.5 °C warming scenarios with three regional climate models (RCMs) in mainland China from 1986 to 2100 by applying the climate-specific exposure-response functions to gridded daily heatwave series and daily mortality across the country.

First, we collected 0.5-degree gridded climate and demographic data in mainland China from 1986 to 2100. Second, we adopted health-related heatwave definition (daily maximum temperature≥ 92.5^th^ with duration ≥3 days), then reanalyzed location-specific exposure-response functions between heatwave and mortality, and combined risks by climate zone through meta-analysis. Third, death burden attributable to heatwaves is derived by applying risk estimates to corresponding gridded heatwave series and mortality across the country, and could be summed to gain provincial and national estimates. Next, to better comprehend the projected health impacts for the future, we decomposed the contribution of driving factors and the uncertainty source of heatwave-related deaths, including heatwave-mortality exposure-response functions, climate effects, population size and aging factors. Finally, we compared the attributable deaths between 1.5 °C warming scenarios and three RCP emission scenarios. Below we showed the detailed methods of calculation and analysis.

**
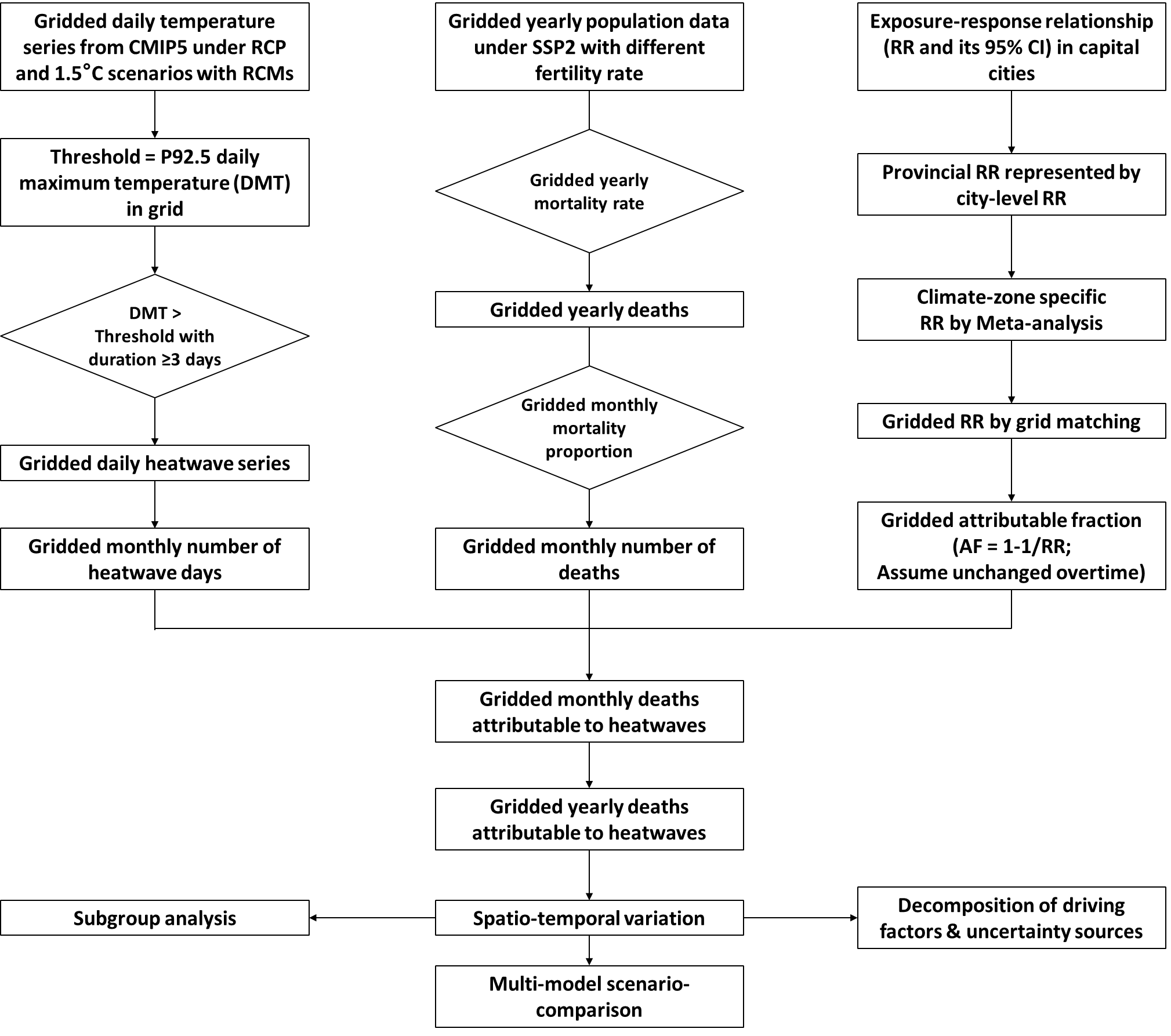
**

**Note: Flowchart of data pre-processing process.** The diamond symbol in the first column indicates whether each day is under a heatwave event according to the pre-selected definition of heatwaves. The diamond symbol in the second column indicates grid-specific yearly mortality rate (or monthly mortality proportion) multiplied by the data in the above rectangle, with the assumption of no changes in mortality rate over time. RCP, representative concentration perspectives; RCM, regional climate models; SSP, shared socioeconomic pathways; CI, confidence interval.

## 1.2 Data collection

### 1.2.1 Projected daily temperature series under climate change scenarios.

***Rationale for using three GCMs to drive RegCM4.4.*** The climate projection data were derived from a regional climate model (RegCM4.4) simulations at a space spacing of 25 km over the CORDEX-East Asia domain. The model was customized for better performance with the updated land cover data to represent the realistic vegetation cover over China.^1-3^ The initial and time evolving lateral boundary conditions needed to drive RegCM4 are derived from three CMIP5 models: HadGEM2-ES, MPI-ESM-MR, and NorESM1-M. We used three different GCMs to drive RegCM4.4 to address the uncertainties.

The three GCMs were selected to drive RegCM4 since (1) data availability (6-hourly data from the surface to the top of the atmosphere), (2) their relatively high resolutions to match the 25 km grid spacing of the RegCM4 (the ratio of the resolution between GCMs and RCMs should not exceed 6-8), (3) their good performance in reproducing present climate over China,^4^ and (4) these GCMs come from different model groups with different structures and roughly cover the range of CMIP5 models’ climate sensitivities, with NorESM1-M (transient climate response, TCR~1.4 ºC) in the low sensitivity group, MPI-ESM-MR (TCR~2.0 ºC) in the medium sensitivity group, and HadGEM2-ES (TCR~2.5 ºC) in the high sensitivity group, as measured by the transient climate response (TCR).^5^

The RegCM4 climate simulations covered the baseline period 1986-2005 (historical simulation) and the future period 2021-2100 (scenario simulations) under three Representative Concentration Pathways (RCP), RCP2.6, RCP4.5 and RCP8.5, to represent low, middle and high emission scenarios, respectively. The three scenarios are most typically used in the RCM community and recommended by the CORDEX frame.

***Rationale for using CMIP5 driven RCM projections.*** Recent CMIP6 GCMs have more comprehensive physical processes than their precedents, but their simulation of climate change over China with complex terrain still has obvious deficiencies.^6,7^ RCMs are commonly used to downscale the coarse resolution GCMs to produce climate variables at spatially high-resolution grids, and also show better performance in reproducing present-day climate over regions. Although it would be good to drive RCM using the most recent CMIP6 outputs, conducting RCM simulations is time-consuming. To conduct RCM simulations, we need to tune the updated RCM version (RegCM5 now for the RegCM series) to customize its performances, select and collect 6-hourly GCM data, conduct the expensive RCM simulations, and bias correct the output, before finally providing the data to impact studies. This time cycle usually takes 3-4 years or more, similar to the cycle of different generations of CMIPs. Therefore, only the available CMIP5 driven RCM projections are used in the present study.

Since the temperature is provided on 0.25° × 0.25° spatial resolution, the model outputs were bias-corrected and downscaled through bi-linear interpolation at a 0.5° × 0.5° resolution and linear interpolated by day of the year.^8,9^ The bias correction and statistical downscaling lead to an improvement of RCMs in reproducing the observed spatial pattern and long-term average of climatic variables.

***Calculation of climate data under the 1.5 °C scenario.*** Following the IPCC definition of 1.5 °C relative to pre-industrial levels,^10^ we calculated the global average temperature rising relative to 1850-1900 for three RCMs. The timing of 1.5 °C is established by a 30-year running average of the global average temperature increase that exceeds 1.5 °C. Under RCP2.6, the global temperature increase reaching 1.5 °C for HadGEM2-ES and MPI-ESM-MR is projected to occur in 2030 (the increase is less than 1.5 °C for NorESM1-M in the 21st century). The temperature in 2020-2039 is used to represent the climate if global warming reaches 1.5 °C.^11,12^

### 1.2.2 Population projections in China

Shared socioeconomic pathways 2 (SSP2) is a dynamic scenario that follows middle-of-the-road pathways and describes moderate challenges to adaptation and mitigation. It assumes medium fertility, mortality, migration rate and education level, and depicts a very likely development scenario for China in the future.^13,14^ Thus, the medium population scenario in our study adopted the assumption of SSP2. Also, high and low population scenarios were derived by assuming high and low fertility rates to reflect the impact of the current fertility policy adjustment.

As for future population changes, the IIASA SSP database’s assumptions of fertility and life expectancy in China are inconsistent with reality, and its population projection provided represents national-level populations, lacking at provincial and grid levels. Whereas Chinese researchers downscaled the provincial projection and established population grids for China considering fertility-promoting policies, which are better in line with the actual situation.^14^ And age-specific future population changes we used were from the latter.

### 1.2.3 Demographic data

Demographic data (e.g. yearly mortality rate and monthly mortality rate) were extracted from Chinese national and provincial statistical yearbooks from 1986 to 2019.

Since the population projection was based on fertility and life expectancy, the future mortality rate is unavailable and we assumed constant mortality rates as in other previous studies, which may also benefit the comparison of their results.^15,16^

Besides, according to Chinese official statistical yearbooks, China’s mortality rate has been relatively stable in the past decade (2010-2019), with slight fluctuations between years. The average value is 7.13‰ (7.09‰ - 7.16‰), which is comparable to the world’s level.^17^ Moreover, we aimed to highlight the role of climate change in the change of attributable deaths, so we assumed that the future mortality rate is the same as in 2010s (2010-2019).

### 1.2.4 Mortality records

The daily mortality data from 2007 to 2013 were collected from the Chinese National Center for Chronic and Non-communicable Disease Control and Prevention, covering 31 provincial capitals in mainland China. The capital cities, which are widely spread across China, have the largest population and high-quality data on causes of death. In general, the mortality data of metropolises are a sound representation of the whole nation.

The underlying cause of death was coded on the basis of the 10th Revision of the International Statistical Classification of Diseases and Related Health Problems (ICD-10), containing non-accidental mortality (ICD-10: A00-R99), mortality due to cardiovascular disease (I00-I99), respiratory disease (J00-J99). The daily number of non-accidental death was further categorized by gender and age group (0-64, 65-74 and 75 years old or above).

## 1.3 Data analysis

### 1.3.1 Selection of health-related heatwave definitions

There is no universally accepted or official definition of a heatwave, so the selection of the most appropriate heatwave definition remains controversial. The definition we used was derived from exposure-response relationship with the best goodness of model fits. Specifically, we compared 15 heatwave definitions combining five heat thresholds and three durations. The Poisson generalized linear model was performed to estimate the effects of heatwave on mortality, and the Q-AIC value was used to assess the goodness of model fits among 15 definitions. The minimal sum of Q-AIC produced the best model fit and optimal definition; therefore, we considered the currently used definition as the best heatwave definition in terms of the ability to capture the real pattern of health effects in view of health risks. And we found that defining a heatwave as at least three consecutive days with daily maximum temperature ≥ 92.5^th^ percentile performed the best model fit.^18^ Since heatwaves almost occur in warm seasons,^19^ we limited our research to May to September. Besides, heatwave days mean each day during heatwave events and we reported the sum of heatwave days for certain years or multi-year averages.

### 1.3.2 Estimation of mortality risks from heatwaves

***Relative risks in each capital city.*** Relative risk (RR) represents the increase in the risk of mortality resulting from heatwave days compared with non-heatwave days. Because of the requirement that all effect sizes of heatwave on mortality should be calculated using a consistent modeling approach, we reanalyzed daily death data of urban residents from 2007 to 2013 derived from Chinese National Center for Chronic and Noncommunicable Disease Control and Prevention, and provided parameter estimates for 31 provincial capital cities in China using a Poisson generalized linear model. The Poisson generalized linear model that allows for over-dispersion, was used to estimate the city-specific mortality risk (relative risk, RR) during heatwave days compared with non-heatwave days. The model is given as follows:

Log(*μ_t_* ) = *α*+ *HW_t_* + *NS*(*Date_t_,4*) +*ηYear_t_* + *NS*(*API_t_,3*) + *γDow_t_* + *νHoliday_t_* + *β_1_RH_t_*,*_l_* + *β_2_PRE_t_*,*_l_*

where *μ_t_* is the expected number of deaths on day *t*; *α* is the intercept; *HW_t_* is the dummy variable assigned 1 for heatwave days and 0 for non-heatwave days; NS() denotes the natural cubic spline function. Day of the season (*Date_t_*) with 4 degrees of freedom (*df*) is used to control for seasonality, and an indicator variable for year (*Year_t_*) is used for the long-term trend of daily mortality. A 3 *df* is applied for the air pollution index (*API_t_*). Public holidays (*Holiday_t_*) and day of the week (*Dow_t_*) are also included in the model as categorical variables. *RH_t_*,*_l_* and *PRE_t_*,*_l_* are the matrices produced by a distributed lag non-linear model with 5 *df* natural cubic spline respectively for relative humidity and atmospheric pressure, and both 4 *df* splines for their lags up to 10 days.

As for the adjustment for air quality to control the impact of air pollution, we considered the air pollution index (API), not specific pollutants. API is an integrated air pollution index generated by a piecewise linear transformation from the concentrations of three air pollutants, including sulfur dioxide (SO_2_), nitrogen dioxide (NO_2_), and fine particulate matter smaller than 10 micrometers (PM_10_).

For additional details on the methodology of acquisition, processing and analysis of data see Yang et al.^18^

***Combination of relative risks in each climate zones.*** Due to the difference in health effects between urban and rural areas, the relationship between heatwave and mortality in different provinces cannot be simply represented by the capital cities in each province. Based on the general trend that risks are homogeneous in the same climate zone and higher in the north of China than that in the south,^20^ the pooled effects of heatwave on mortality at 31 cities across seven climate zones were produced using a meta-analysis to identify regional patterns of mortality risk in response to heatwaves.

After we obtained the relative risk of each capital city, the pooled effects of heatwave on mortality at 7 climate zones across 31 cities were produced using a meta-analysis. The selection of random effect or fixed effect meta-analysis was based on Cochran’s Q test and I^2^ statistic, which quantified the proportion of variability due to the true differences across cities. When the Q test yielded statistically significant results and I^2^ ≥ 50%, the random effect model based on restricted maximum likelihood estimation (REML) was applied. Otherwise, the fixed effect model was used.^21,22^ The process and equations are attached as below.

The random-effects model can be formulated as:

*T*_i_ =*μ*_θ_+ *u*_i_ +*e*_i_,

where *T*_i_ represents the *i*th effect estimate in a collection of *k* studies (*i* = 1, 2, ..., *k*) (the 31 different cities were considered as *k* studies), *μ*_θ_ represents the mean value of parametric effect sizes θ_i_. The errors *u*_i_ and *e*_i_ represent the two variability sources affecting the effect estimates, *T*_i_; and quantified by the between-studies, τ^2^, and within-study, σ_i_^2^, variances. Specifically, the conditional variance, σ_i_^2^, represents the within study variability, that is, the variability produced by random sampling. The unconditional variance, τ^2^, represents the extent of true heterogeneity among the study effects produced by the influence of substantive (e.g., population age structure, economic level, etc.) and methodological (e.g., sample size) characteristics of the studies. *T*_i_ ~ N(*μ*_θ_, τ^2^+σ_i_^2^).

When there is assumed no heterogeneity among the effect estimates, then the between-studies variance is zero (τ^2^ = 0), and there only will be variability due to sampling error, σ_i_^2^. In this case, all the studies estimate one parametric effect size, θ_i_ = θ, and the statistical model simplifies to *T*_i_ = θ +*e*_i_, thus becoming a fixed-effects model. So, the fixed-effects model can be considered as a particular case of the random-effects model when there is no between-studies variability and, as a consequence, the effect estimates, *T*_i_, are only affected by sampling error, σ_i_^2^. *T*_i_ ~ N(θ, σ_i_^2^).

Here we can simply think that the random effect term is the individuality of the city; while in the fixed effect model, the effects in all cities are considered the same and there is no difference between cities.

Forest plots visually showing the effect sizes (RR) from individual studies in 31 provinces and the aggregate effect from a meta-analysis in 7 climate zones of China are shown as Figure S2.

Then, we got gridded *RR_p_* in grid *p* by matching climate division-specific RR through meta-analysis with the grid in different climate zones.

***Relative risks in subgroup population.*** We provided parameters on exposure-response function of the subgroup population *g* with different gender, age groups and underlying diseases in different regions under the health-related heatwave definition.

*RR_p,g_* refers to the grid cell-level relative risk caused by different subgroup *g*-specific characteristics (e.g. in a population with certain disease, or certain age, or different gender) in grid *p*. The method we estimated *RR_p,g_* was similar to the way we estimated *RR_p_*. The difference between them is that we replaced the outcome with subgroup *g*-specific deaths data when estimating *RR_p,g_*.

As for the death outcome, the daily death data were derived from NCNCD, and the underlying cause of death was on the basis of ICD codes, containing non-accidental mortality (ICD-10: A00-R99), mortality due to cardiovascular disease (I00-I99) and respiratory disease (J00-J99). The daily number of deaths was further categorized by gender (male or female), age group (0-64, 65-74 and ≥75 years old), respectively. By this way, we can obtain the death data of different diseases, sex, and age group, and calculate *RR_g_* in subgroup *g* in each capital city. Then we used meta-analysis to combine *RR_g_* in different climate zones and matched them with grid in corresponding climate zones and gained *RR_p,g_*.

The exposure-response relationship is assumed to be consistent during the study period, thus we derived *RR_y,p,g_* and *RR_y,p_* from *RR_p,g_* and *RR_p_*.

The boundaries for the seven climate zones in China were derived from Zheng et al.^23^ Administrative boundaries were downloaded from the National Geographic Information Public Service Platform of China.

### 1.3.3 Calculation of heatwave-attributable deaths

The attributable deaths (AN) to a heatwave are the number of deaths during the heatwave period multiplied by the attributable fraction of the heatwave. The model is as follows:

$${AN}_{y, p}=\sum^{m} {Pop}_{y, p}\times{Mort}_{d,m,y,p}\times{HW}_{m,y,p}\times{AF}_{y,p}$$

where *Pop_y, p_* refers to the grid cell-level population size in year *y* and in grid *p*, which is derived from yearly population projections.*Mort*_y,_ *_p_* is the yearly baseline mortality rate. Data on the demographic characteristics (e.g. yearly mortality rate, monthly mortality proportion) of China’s population in each province from 1986 to 2019 were extracted from the Chinese national and provincial statistical yearbooks, which is accurate down to the provincial level and further matched with 0.5 degrees grid to get gridded demographic data. *Mort_y,p_* is multiplied by the monthly mortality proportion then divided by days in month *m* to obtain the daily mortality rate *Mort_d,m,y,p_* in month *m* of year *y*_._ We assumed the mortality rate to be unchanged from 2020 until the end of the century, that is to say, the average value of mortality rates in the 2010s is regarded as the baseline mortality rate for the future. *HW_m,y,p_* is the heatwave days in month *m* of year *y* in grid *p*, which is derived from climate projections under different emission scenarios and climate models. *AF_y,p_* is the attributable fraction of heatwave, which is calculated as:

$${AF}_{y,p}=({RR}_{y,p}-1)/{RR}_{y,p}$$

where *RR_y, p_* refers to the gridded relative risk (RR) in year *y* and in grid *p*. RR in provincial capital cities was derived from real-world mortality and heatwave data from 2003 to 2007. Then the provincial RR is represented by city-level RR, which is delivered to derive climate-zone specific RR by meta-analysis. We also provided parameters on exposure-response function of the subgroup population with different gender, age groups and underlying diseases in different regions. Then we matched the regional statistics with 0.5 degrees grid to get gridded RR data. We assumed the RR values are unchanged overtime.

To identify the health effects of heatwaves among vulnerable subpopulations, we separately calculated AN by causes of gender, age, and disease categories. AN in a given year *y* can be calculated as:

$${AN}_{y, p,g}=\sum^{m} {Pop}_{y, p}\times{Mort}_{d,m,y,p,g}\times{HW}_{m,y,p}\times{AF}_{y,p,g}$$

Where *Pop_y,p_*, *HW_m,y,p_*are the same described in the main text; *Mort_d,m,y,p,g_* is the subgroup *g*-specific daily mortality rate, which is *Mort_d,m,y,p_* multiplied by the death proportion of a certain subgroup *g*; *AF_y, p, g_* refers to the proportion of deaths attributed to heatwaves, caused by different subgroup characteristics (e.g. in a population with certain disease, or certain age, or different gender).

### 1.3.4 Decomposing the drivers of heatwave-attributable deaths

We dissected the contributions of driving factors including heatwave exposure, population growth, and age structure, to the change in attributable deaths to heatwaves using the decomposition method.^24^ The data source (e.g. heatwave days, population size, and population structure) is from the climate and population change projection data during 1986-2100 we used in this study.

This approach estimated the contribution of different factors by sequentially introducing each factor into the AN equation. We assumed that the future mortality rate is the same as in 2010s. The difference between each consecutive step provided an estimate of the relative contribution of each factor. Notably, the order in which each factor is included can influence the results. That is to say, if the sequence of adding factors is not considered, a large bias may occur. Thus, we estimated the results under all sequence permutations (a total of 6 possible sequences) of the three factors. The final estimation of contributions from different factors was the average of the results for all sequences. Moreover, the driving factors of certain regions in different periods were also examined. Detailed equations and processes were shown in the figure and note below.


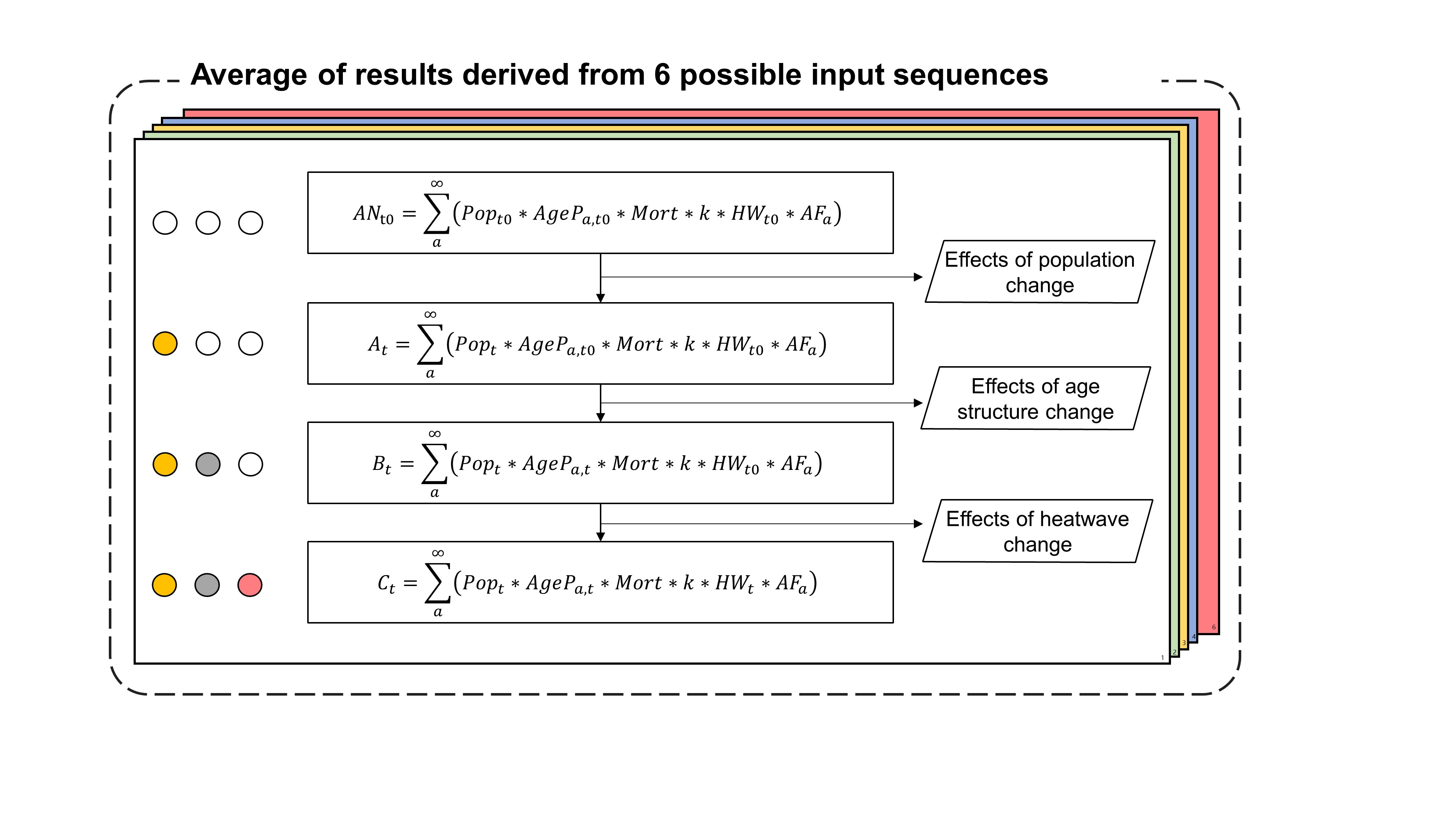


**Note: Framework of the decomposition analysis.** The matrix of colored dots indicates specific combinations of driving factors. Yellow, gray, and red dots refer to the changes in population size, age structure, and heatwave, respectively. *t_0_* and *t* refer to the baseline period and target period. (For example, when we decompose the relative contribution of drivers contributing to changes in attributable deaths from 2030 to 2060, the baseline period is 2030 and the target period is 2060). AN*_t0_* is the annual attributable deaths to heatwaves in the baseline period, which were calculated based on the factors in the baseline period. A*_t_* and B*_t_* are the intermediate variables, which consider the changes in population and age structure incrementally from the baseline period to target period. C*_t_*, is the annual attributable deaths to heatwaves in the target period, which consider all the changes in three factors. In detailed equations, AF*_a_* refers to the age-specific proportion of deaths attributed to heatwaves; Pop refers to the total population; Mort is the mortality rate; HW is the number of heatwave days; AgeP*_a_* is the proportion of the population with age *a*. Besides, we assume that AF is constant during 1986-2100.

### 1.3.5 Uncertainty in research

The uncertainties in this research should be noted, which mainly come from temporal and spatial assumptions in modeling. First, if daily data are unavailable, we assume that the situation keeps the same for a period of time, that is, monthly or annual data are used instead. Besides, if future data are unavailable, we assume that the situation remains unchanged till the end of this century if the variables are stable in the past decade. Second, if gridded data are unavailable, we assume that the situation is the same for each grid in a region, that is, province or region-level data are used to represent each grid within its scope.

# 2. Supplementary Results

**Table S1.** **Heatwave-attributable deaths number under climate and population change scenarios in China.**

| **RCP** | **Time** | **AN (95% CI, *1000)** | | | | | |
| --- | --- | --- | --- | --- | --- | --- | --- |
|  |  | **S1** | | **S2** | | **S3** | |
| RCP2.6 | Baseline | **10.264** | (5.996, 14.094) | **10.264** | (5.996, 14.094) | **10.264** | (5.996, 14.094) |
|  | 2020 | **26.236** | (11.981, 48.648) | **26.328** | (12.023, 48.829) | **26.269** | (11.995, 48.713) |
|  | 2030 | **28.912** | (13.575, 52.495) | **29.171** | (13.682, 52.988) | **29.098** | (13.645, 52.860) |
|  | 2040 | **30.692** | (14.366, 53.492) | **31.225** | (14.596, 54.474) | **31.334** | (14.660, 54.661) |
|  | 2050 | **30.381** | (14.367, 47.523) | **31.278** | (14.767, 48.947) | **31.636** | (14.930, 49.510) |
|  | 2060 | **27.746** | (13.579, 44.159) | **29.086** | (14.200, 46.346) | **29.771** | (14.455, 47.492) |
|  | 2070 | **24.772** | (12.035, 40.439) | **26.544** | (12.849, 43.350) | **27.640** | (13.371, 45.131) |
|  | 2080 | **21.164** | (9.902, 32.541) | **23.392** | (10.933, 36.048) | **24.961** | (11.661, 38.535) |
|  | 2090 | **17.788** | (8.043, 29.849) | **20.303** | (9.153, 34.179) | **22.215** | (9.964, 37.454) |
| RCP4.5 | Baseline | **10.264** | (5.996, 14.094) | **10.264** | (5.996, 14.094) | **10.264** | (5.996, 14.094) |
|  | 2020 | **20.975** | (11.747, 31.562) | **21.056** | (11.793, 31.688) | **21.007** | (11.766, 31.609) |
|  | 2030 | **26.869** | (14.972, 39.951) | **27.121** | (15.103, 40.322) | **27.065** | (15.077, 40.244) |
|  | 2040 | **30.389** | (15.516, 46.379) | **30.918** | (15.771, 47.196) | **31.036** | (15.819, 47.386) |
|  | 2050 | **33.802** | (15.936, 55.040) | **34.849** | (16.399, 56.772) | **35.282** | (16.605, 57.471) |
|  | 2060 | **36.683** | (18.424, 59.560) | **38.475** | (19.238, 62.470) | **39.399** | (19.630, 63.963) |
|  | 2070 | **35.022** | (17.270, 58.038) | **37.558** | (18.470, 62.427) | **39.138** | (19.211, 65.166) |
|  | 2080 | **31.747** | (14.713, 55.927) | **35.134** | (16.189, 62.023) | **37.535** | (17.240, 66.356) |
|  | 2090 | **30.608** | (14.803, 52.421) | **35.025** | (16.834, 59.865) | **38.409** | (18.380, 65.654) |
| RCP8.5 | Baseline | **10.264** | (5.996, 14.094) | **10.264** | (5.996, 14.094) | **10.264** | (5.996, 14.094) |
|  | 2020 | **24.853** | (13.789, 38.418) | **24.932** | (13.831, 38.535) | **24.880** | (13.800, 38.463) |
|  | 2030 | **28.840** | (14.583, 48.038) | **29.094** | (14.701, 48.474) | **29.027** | (14.663, 48.380) |
|  | 2040 | **34.874** | (17.764, 56.785) | **35.479** | (18.024, 57.757) | **35.609** | (18.102, 57.954) |
|  | 2050 | **42.760** | (22.584, 66.737) | **44.086** | (23.251, 68.872) | **44.627** | (23.515, 69.724) |
|  | 2060 | **49.864** | (24.634, 84.352) | **52.341** | (25.779, 88.601) | **53.630** | (26.380, 90.823) |
|  | 2070 | **57.359** | (27.812, 98.892) | **61.626** | (29.813, 106.527) | **64.316** | (31.081, 111.308) |
|  | 2080 | **62.570** | (30.217, 105.110) | **69.317** | (33.325, 116.712) | **74.101** | (35.543, 124.975) |
|  | 2090 | **63.085** | (29.926, 103.950) | **72.259** | (33.911, 119.394) | **79.292** | (37.183, 130.936) |

The number of heatwave-attributable deaths by time, RCP, and population scenario. Baseline refers to the baseline period (1986-2005). 20-year-average attributed deaths for the whole nation are represented as 2020 (2011-2030), 2030 (2021-2040), …, to 2090 (2081-2100). Monte Carlo simulations generating 1000 samples were computed to produce the empirical CI. RCP, representative concentration perspectives; AN, number of deaths attributable to heatwaves; CI, confidence interval. S1/2/3, low/medium/high population scenarios.

**Table S2. Percentage change of heatwave-attributable deaths under climate and population change scenarios in China.**

| **RCP** | **Time** | **Percentage change (95% CI, %)** | | | | | | | |
| --- | --- | --- | --- | --- | --- | --- | --- | --- | --- |
|  |  | **S1** | |  | **S2** | |  | **S3** | |
| RCP2.6 | Baseline | - | |  | - | |  | - | |
|  | 2020 | **156.1** | (71.1, 299.3) |  | **157.0** | (71.7, 300.7) |  | **156.4** | (71.3, 299.8) |
|  | 2030 | **182.2** | (89.5, 333.2) |  | **184.8** | (91.1, 337.4) |  | **184.0** | (90.6, 336.3) |
|  | 2040 | **199.6** | (106.2, 343.8) |  | **204.8** | (109.7, 351.9) |  | **205.9** | (110.4, 353.5) |
|  | 2050 | **196.7** | (117.0, 277.3) |  | **205.4** | (123.6, 288.5) |  | **208.9** | (126.2, 293.0) |
|  | 2060 | **170.9** | (95.1, 264.2) |  | **184.0** | (104.2, 282.7) |  | **190.7** | (108.8, 292.1) |
|  | 2070 | **141.8** | (79.2, 238.6) |  | **159.0** | (91.8, 262.6) |  | **169.7** | (99.9, 277.1) |
|  | 2080 | **106.5** | (51.2, 166.6) |  | **128.3** | (67.2, 195.1) |  | **143.6** | (79.0, 215.2) |
|  | 2090 | **73.6** | (20.5, 152.9) |  | **98.2** | (37.1, 189.0) |  | **116.9** | (49.6, 216.3) |
| RCP4.5 | Baseline | - | |  | - | |  | - | |
|  | 2020 | **104.7** | (66.6, 151.7) |  | **105.5** | (67.3, 152.7) |  | **105.1** | (66.9, 152.1) |
|  | 2030 | **162.1** | (117.8, 212.6) |  | **164.5** | (119.8, 215.5) |  | **164.0** | (119.4, 214.9) |
|  | 2040 | **196.4** | (127.0, 268.2) |  | **201.6** | (130.4, 275.0) |  | **202.8** | (131.3, 276.5) |
|  | 2050 | **229.9** | (139.0, 337.9) |  | **240.2** | (146.4, 351.6) |  | **244.4** | (149.6, 357.1) |
|  | 2060 | **258.1** | (182.7, 378.3) |  | **275.6** | (196.0, 402.4) |  | **284.6** | (202.9, 414.8) |
|  | 2070 | **241.9** | (156.9, 368.8) |  | **266.7** | (175.0, 403.3) |  | **282.1** | (185.3, 424.9) |
|  | 2080 | **210.2** | (113.6, 353.1) |  | **243.2** | (135.0, 402.6) |  | **266.7** | (150.3, 437.1) |
|  | 2090 | **198.9** | (116.7, 326.9) |  | **242.1** | (147.6, 388.2) |  | **275.1** | (170.5, 435.4) |
| RCP8.5 | Baseline | - | |  | - | |  | - | |
|  | 2020 | **142.6** | (92.5, 206.2) |  | **143.4** | (93.2, 207.2) |  | **142.9** | (92.7, 206.5) |
|  | 2030 | **181.7** | (115.7, 289.3) |  | **184.1** | (117.5, 293.0) |  | **183.5** | (117.0, 292.2) |
|  | 2040 | **240.5** | (161.3, 359.4) |  | **246.4** | (165.5, 367.3) |  | **247.7** | (166.5, 368.9) |
|  | 2050 | **317.4** | (230.9, 437.9) |  | **330.4** | (240.1, 455.4) |  | **335.6** | (244.1, 462.4) |
|  | 2060 | **387.2** | (251.3, 586.4) |  | **411.4** | (267.6, 621.4) |  | **424.0** | (276.7, 639.5) |
|  | 2070 | **460.5** | (291.9, 706.2) |  | **502.2** | (319.8, 767.4) |  | **528.5** | (337.4, 805.2) |
|  | 2080 | **511.1** | (341.8, 754.0) |  | **577.0** | (386.7, 847.1) |  | **623.7** | (419.6, 913.4) |
|  | 2090 | **516.1** | (337.8, 745.5) |  | **605.7** | (399.8, 869.0) |  | **674.3** | (447.1, 962.6) |

The percentage change of heatwave-attributable deaths by time, RCP, and population scenario. Baseline refers to the baseline period (1986-2005). 20-year-average attributed deaths for the whole nation are represented as 2020 (2011-2030), 2030 (2021-2040), … to 2090 (2081-2100). Monte Carlo simulations generating 1000 samples were computed to produce the empirical CI. RCP, representative concentration perspectives; CI, confidence interval. S1/2/3, low/medium/high population scenarios.

**Table S3. Uncertainty source of changes in attributable deaths.**

| **Time** | **Climate change scenarios** | **Population scenarios (%)** | **ERR parameters (%)** | **Climate models (%)** |
| --- | --- | --- | --- | --- |
| 2030 | RCP2.6 | 0.4 | 80.4 | 19.3 |
|  | RCP4.5 | 0.4 | 85.0 | 14.6 |
|  | RCP8.5 | 0.4 | 76.2 | 23.4 |
|  | mean | 0.4 | 80.5 | 19.1 |
| 2060 | RCP2.6 | 4.0 | 76.2 | 19.8 |
|  | RCP4.5 | 4.0 | 77.1 | 18.9 |
|  | RCP8.5 | 4.0 | 77.0 | 19.1 |
|  | mean | 4.0 | 76.8 | 19.3 |
| 2090 | RCP2.6 | 11.7 | 70.2 | 18.1 |
|  | RCP4.5 | 11.4 | 68.7 | 19.9 |
|  | RCP8.5 | 11.8 | 74.0 | 14.2 |
|  | mean | 11.6 | 71.0 | 17.4 |
| **Time average** |  | **5.3** | **76.1** | **18.6** |

RCP, representative concentration perspectives; ERR, exposure-response relationship. The last line is the average proportion of the sources of uncertainty over time.

**Table S4. Heatwave-attributable deaths under** **1.5 °C and three RCP scenarios in 2090.**

| **Region** | **1.5 °C** | | **Difference** | | | | | |
| --- | --- | --- | --- | --- | --- | --- | --- | --- |
|  |  |  | **RCP2.6** | | **RCP4.5** | | **RCP8.5** | |
|  | **AN** | **%** | **AN** | **%** | **AN** | **%** | **AN** | **%** |
| Central | 3927 | 23.4 | 681 | 19.3 | 3897 | 21.3 | 13056 | 23.5 |
| East | 4720 | 28.1 | 691 | 19.6 | 4665 | 25.6 | 14899 | 26.8 |
| North | 1963 | 11.7 | 928 | 26.3 | 3869 | 21.2 | 8242 | 14.9 |
| Northeast | 293 | 1.7 | 68 | 1.9 | 573 | 3.1 | 1312 | 2.4 |
| Northwest | 1476 | 8.8 | 471 | 13.3 | 2507 | 13.7 | 6407 | 11.5 |
| South | 2552 | 15.2 | 516 | 14.6 | 786 | 4.3 | 5831 | 10.5 |
| Southwest | 1838 | 11 | 179 | 5.1 | 1960 | 10.7 | 5745 | 10.4 |
| **Nation** | **16769** | **100** | **3534** | **100** | **18257** | **100** | **55492** | **100** |

Proportion (%) refers to the proportion of the attributable deaths in certain regions to the whole country. Difference denotes the difference between 1.5 °C and three RCP scenarios in 2090, which refers to the avoidable deaths under the 1.5 °C scenario. RCP, representative concentration perspectives; AN, number of deaths attributable to heatwaves.

**

**

**Fig S1. Bias correction of the modeled temperature series under different scenarios.**

Comparison between the distribution (left panel) and cumulative distribution (right panel) of the raw and bias-corrected modeled maximum temperature and the observed maximum temperature series for the period of 1986-2100 in grid of 134.75°E, 47.75°N in Heilongjiang province, Northeast China. RCM indicates regional circulation model, namely had (MOHC-HadGEM2-ES), mpi (MPI-ESM- MR), and nor (NorESM1-M) in this study.


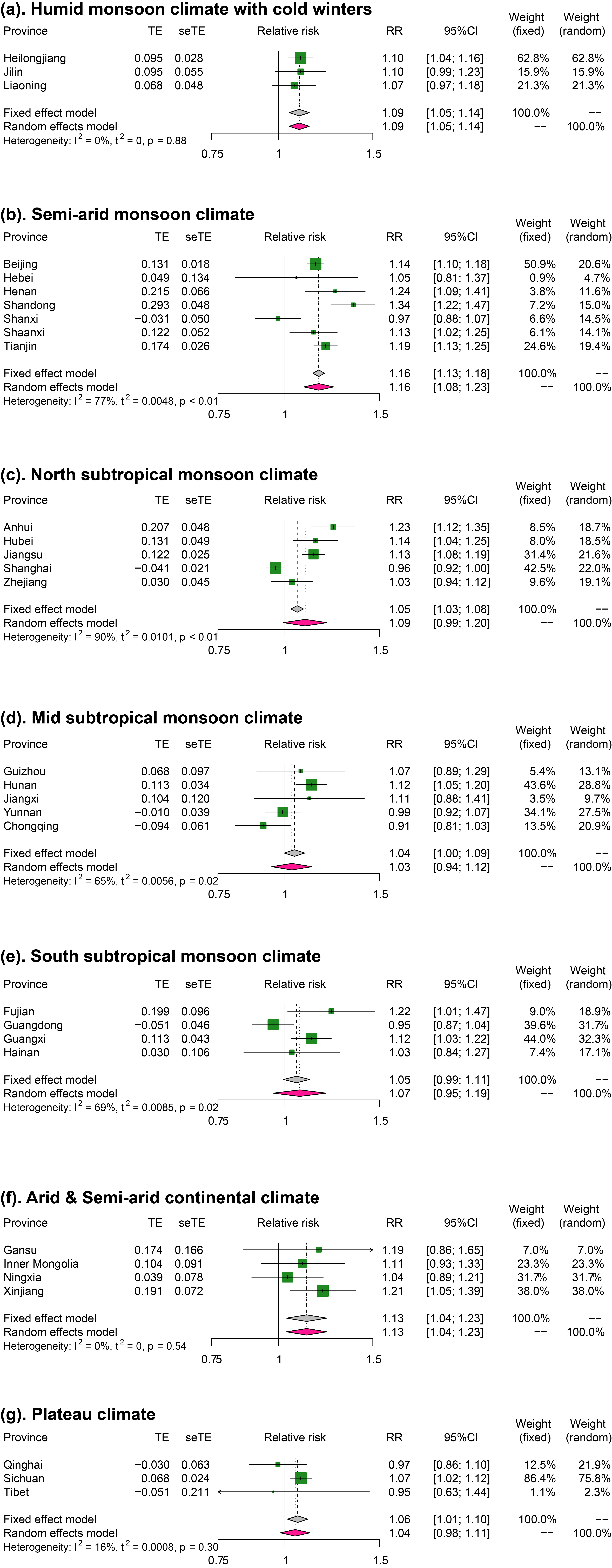


**Fig S2. Forest plots of the relative risks of heatwave-related mortality in different climate zones of China.**

Forest plots show the effect sizes (relative risks, RR) from individual studies in 31 provinces and the aggregate effect from a meta-analysis in 7 climate zones. Square indicates point estimate of each province. Size of square indicates the relative contribution of each province. Solid horizontal line represents 95% confidence intervals (CI) of each province. The vertical reference line indicates the point on the x-axis equals to no effect. Dashed vertical line represents the pooled value for each climate zone from fixed effect model. Dotted vertical line represents the pooled value for each climate zone from random effect model. At the bottom of each panel, a diamond shape represents overall effect. The length of the diamond symbolizes 95% CI of the pooled result. TE is the estimate of treatment effect, which refers to log relative risk in this study; and seTE is the standard error of treatment estimate.

**
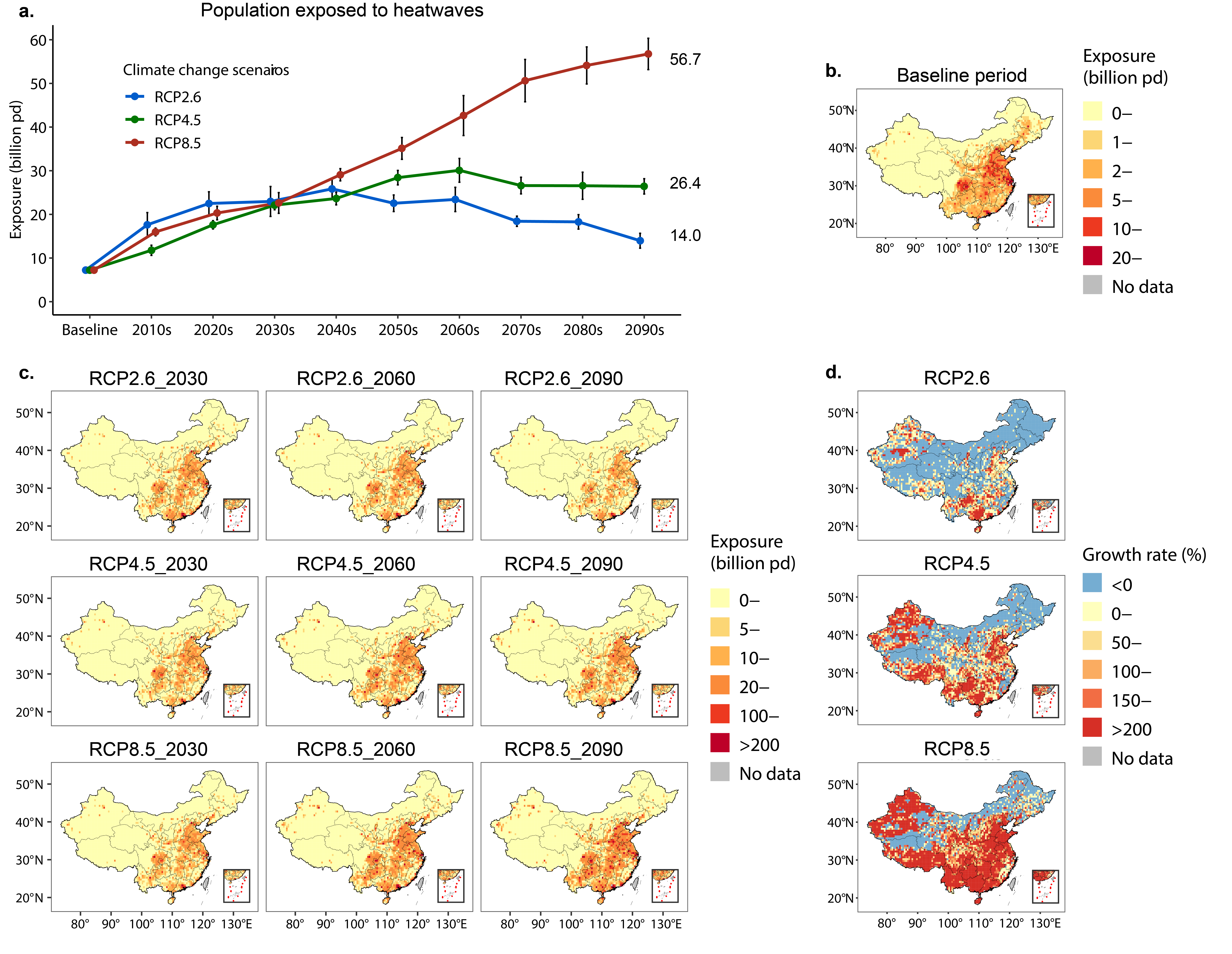
**

**Fig S3. Projections of** **decade-average population exposure to heatwaves in China.**

**a**, Temporal trends of national decade-average population exposed to heatwaves under three RCP scenarios from 1986 to 2100. Solid lines denote the estimated mean decade-average population exposure (person-days) across the climate models. The solid vertical black line represents the 95% confidence interval of the ensemble of models. Baseline refers to the baseline period (1986-2005). **b**, Spatial differentiation of multi-years-average population exposure in 0.5 degrees grid during the baseline period. **c**, Spatial differentiation of multi-years-average population exposure in grid in 2030 (2021-2040), 2060 (2051-2070), 2090 (2081-2100). **d**, Changes in population exposure in 2090 (2081-2100) relative to the baseline period. Growth rate (%) = (Exposure_2090_- Exposure_baseline_) / Exposure_baseline_×100%. RCP, representative concentration perspectives; pd, person-days.

**
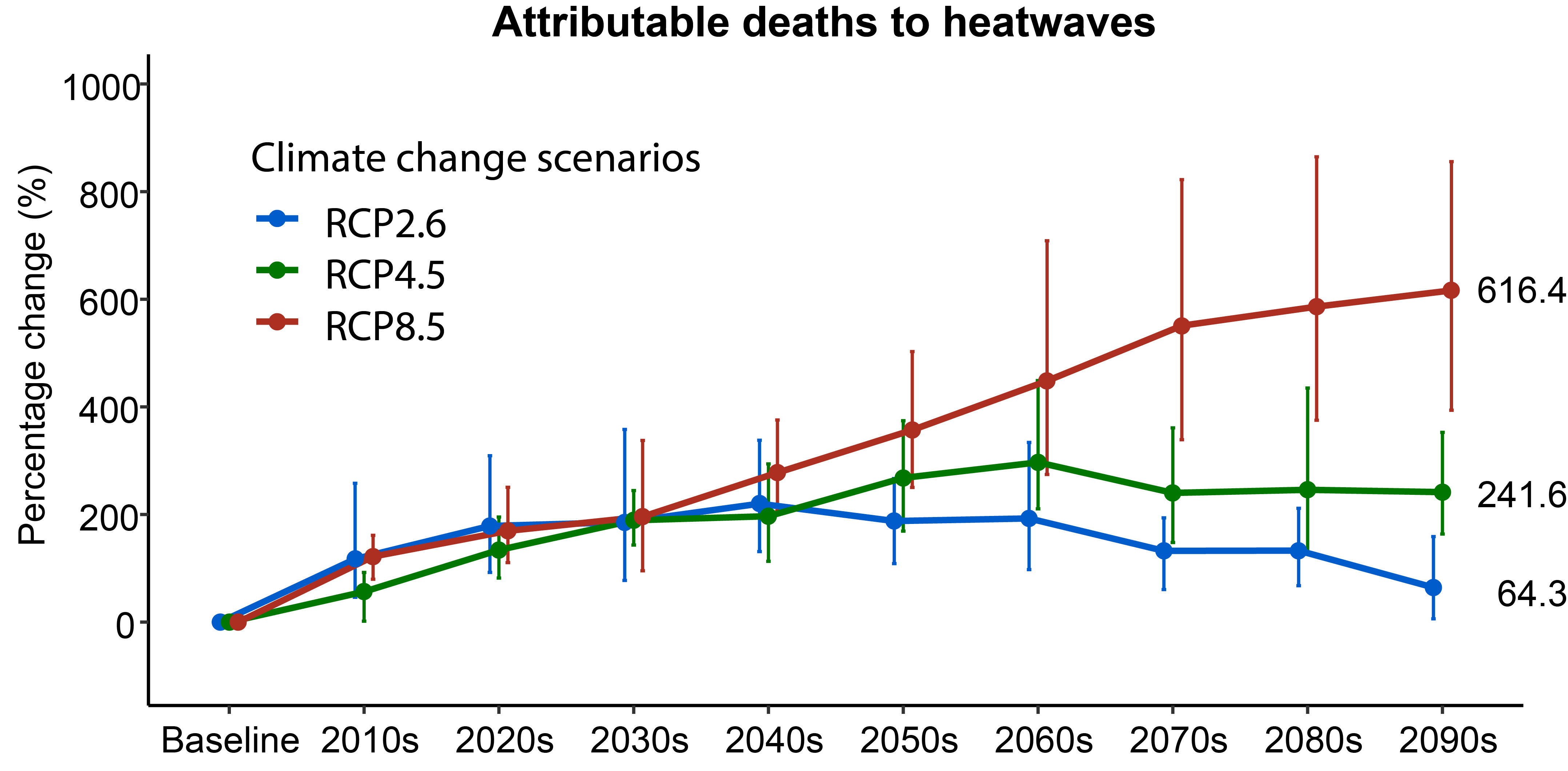
**

**Fig S4. Temporal trends of percentage change on national decade-average heatwave-attributed deaths under three RCP scenarios from 1986 to 2100.**

Solid lines denote the estimated mean decade-average attributed deaths across the three climate models with middle-fertility assumption. The solid vertical line represents the 95% confidence interval of the ensemble of models. Baseline refers to the baseline period (1986-2005). The number marked on the right is the average attributable deaths from 2090 to 2099. Monte Carlo simulations generating 1000 samples were computed to produce the empirical CI. The percentage change of death toll would continue to rise under RCP8.5, but turn down from 2040-2060 under RCP2.6 and RCP4.5. By the end of the century, the mortality from heatwaves is projected to increase by 64.3%, 241.6%, 616.4%, under RCP2.6, RCP4.5 and RCP8.5, respectively, compared to that in the baseline period.

**
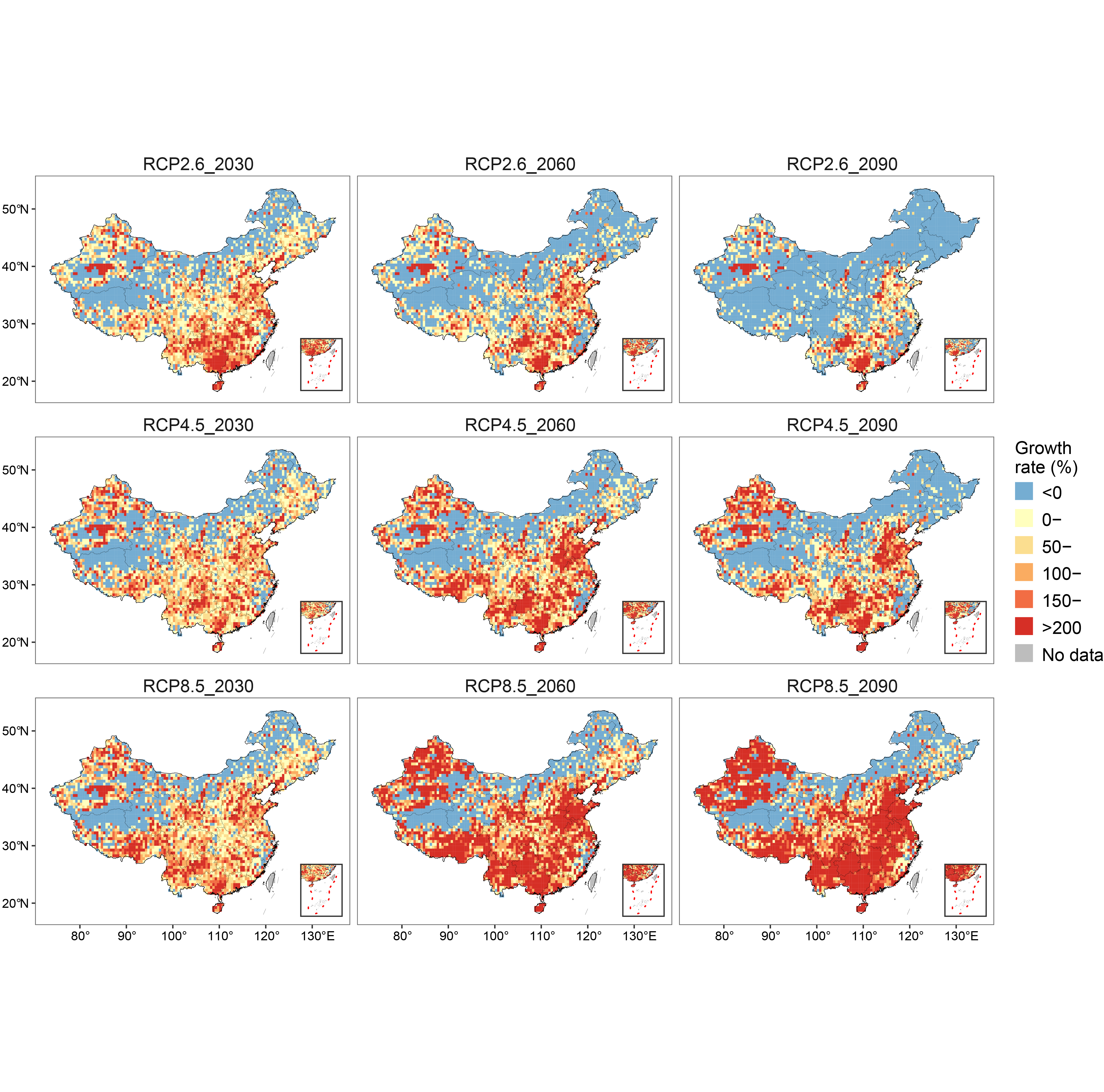
**

**Fig S5. Changes in attributable deaths to heatwaves in 2030, 2060, 2090 relative to the baseline period in China.**

Percentage changes in attributable deaths to heatwaves in 2030 (2021-2040), 2060 (2051-2070), and 2090 (2081-2100) relative to the baseline period with middle-fertility assumption. Growth rate (%) = (AN_target year_- AN_baseline_) / AN_baseline_×100%. RCP, representative concentration perspectives; AN, number of deaths attributable to heatwaves. Under RCP2.6 scenario, the growth rate in 2030 compared with the baseline period is higher than that in 2060 and 2090, which is mainly due to the declining trend of absolute attributable deaths after 2040-2050. Spatially, the number of attributable deaths showed an increasing trend almost in all provinces except northeast areas, and the increase was larger in south and southwest China.


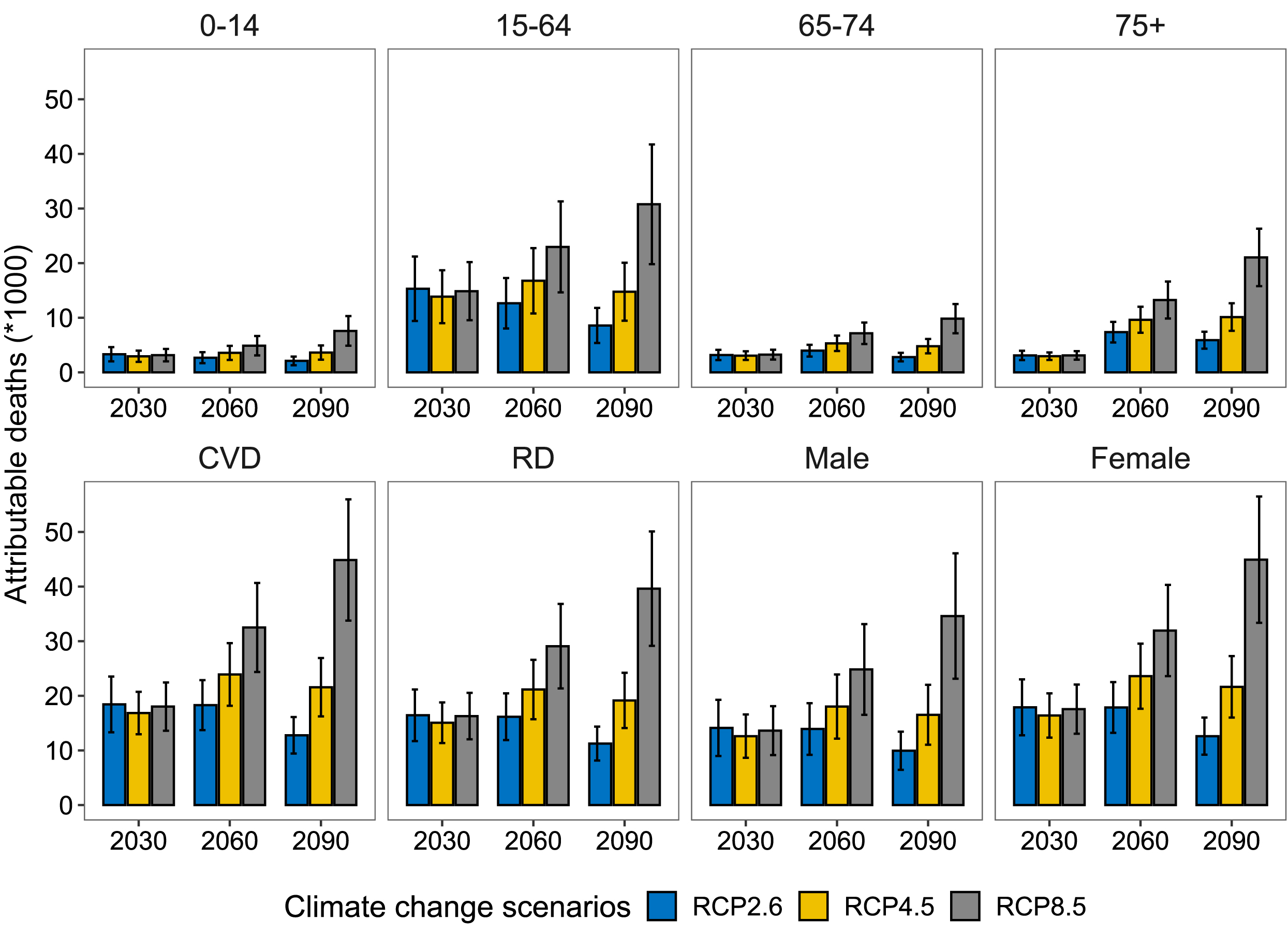


**Fig S6. Multi-year average heatwave-attributable deaths in different groups in 2030, 2060, 2090 under three climate change scenarios.**

2030, 2060, 2090 refer to the periods of 2021-2040, 2051-2070, 2081-2100, respectively. CI, confidence interval; CVD, cardiovascular diseases; RD, respiratory diseases. The solid vertical black line represents the 95% confidence intervals (CI) of the ensemble of models.

# References:

1. Giorgi F, Coppola E, Solmon F, et al. RegCM4: model description and preliminary tests over multiple CORDEX domains. *Climate Research* 2012; **52**: 7-29.

2. Gao X, Shi Y, Giorgi F. Comparison of convective parameterizations in RegCM4 experiments over China with CLM as the land surface model. *Atmospheric and Oceanic Science Letters* 2016; **9**(4): 246-54.

3. Gao X, Shi Y, Han Z, et al. Performance of RegCM4 over major river basins in China. *Advances in Atmospheric Sciences* 2017; **34**(4): 441-55.

4. Jiang D, Tian Z, Lang X. Reliability of climate models for China through the IPCC Third to Fifth Assessment Reports. *International Journal of Climatology* 2016; **36**(3): 1114-33.

5. Chen X, Guo Z, Zhou T, et al. Climate Sensitivity and Feedbacks of a New Coupled Model CAMS-CSM to Idealized CO2 Forcing: A Comparison with CMIP5 Models. *Journal of Meteorological Research* 2019; **33**(1): 31-45.

6. Xin XG, Wu TW, Zhang J, Yao JC, Fang YJ. Comparison of CMIP6 and CMIP5 simulations of precipitation in China and the East Asian summer monsoon. *International Journal of Climatology* 2020; **40**(15): 6423-40.

7. Wu J, Shi Y, Xu Y. Evaluation and Projection of Surface Wind Speed Over China Based on CMIP6 GCMs. *Journal of Geophysical Research-Atmospheres* 2020; **125**(22).

8. Hempel S, Frieler K, Warszawski L, Schewe J, Piontek F. A trend-preserving bias correction the ISI-MIP approach. *Earth System Dynamics Discussions* 2013; **4**: 219-36.

9. Vicedo-Cabrera AM, Sera F, Gasparrini A. Hands-on Tutorial on a Modeling Framework for Projections of Climate Change Impacts on Health. *Epidemiology* 2019; **30**(3): 321-9.

10. IPCC. Global Warming of 1.5 C.An IPCC Special Report on the impacts of global warming of 1.5 C above pre-industrial levels and related global greenhouse gas emission pathways, in the context of strengthening the global response to the threat of climate change, sustainable development, and efforts to eradicate poverty, 2018.

11. Warszawski L, Frieler K, Huber V, Piontek F, Serdeczny O, Schewe J. The Inter-Sectoral Impact Model Intercomparison Project (ISI-MIP): project framework. *Proc Natl Acad Sci U S A* 2014; **111**(9): 3228-32.

12. Su B, Huang J, Fischer T, et al. Drought losses in China might double between the 1.5 degrees C and 2.0 degrees C warming. *Proc Natl Acad Sci U S A* 2018; **115**(42): 10600-5.

13. O’Neill BC, Kriegler E, Ebi KL, et al. The roads ahead: Narratives for shared socioeconomic pathways describing world futures in the 21st century. *Global Environmental Change* 2017; **42**: 169-80.

14. Chen Y, Guo F, Wang J, Cai W, Wang C, Wang K. Provincial and gridded population projection for China under shared socioeconomic pathways from 2010 to 2100. *Sci Data* 2020; **7**(1): 83.

15. Guo Y, Gasparrini A, Li S, et al. Quantifying excess deaths related to heatwaves under climate change scenarios: A multicountry time series modelling study. *PLoS Med* 2018; **15**(7): e1002629.

16. Yang J, Zhou M, Ren Z, et al. Projecting heat-related excess mortality under climate change scenarios in China. *Nature Communications* 2021; **12**(1): 1039.

17. National Bureau of Statistics of the People's Republic of China. China Statistical Yearbook: China Statistics Press; 2019.

18. Yang J, Yin P, Sun J, et al. Heatwave and mortality in 31 major Chinese cities: Definition, vulnerability and implications. *Sci Total Environ* 2019; **649**: 695-702.

19. Vaidyanathan A, Malilay J, Schramm P, Saha S. Heat-Related Deaths — United States, 2004–2018. *Morbidity and Mortality Weekly Report* 2020; **69**(24): 729-34.

20. Chen R, Yin P, Wang L, et al. Association between ambient temperature and mortality risk and burden: time series study in 272 main Chinese cities. *BMJ (Online)* 2018; **363**: k4306-k.

21. Huedo-Medina TB, Sanchez-Meca J, Marin-Martinez F, Botella J. Assessing heterogeneity in meta-analysis : Q statistic or I2 index? *Psychological methods* 2006; **11**(2): 193-206.

22. Viechtbauer W. Conducting Meta-Analyses in R with the metafor Package. *Journal of statistical software* 2010; **36**(3): 1-48.

23. Zheng J, Yin Y, Li B. A New Scheme for Climate Regionalization in China. *Acta Geographica Sinica* 2010; **65**(01): 3-12.

24. Stanaway JD, Afshin A, Gakidou E, et al. Global, regional, and national comparative risk assessment of 84 behavioural, environmental and occupational, and metabolic risks or clusters of risks for 195 countries and territories, 1990–2017: a systematic analysis for the Global Burden of Disease Study 2017. *The Lancet* 2018; **392**(10159): 1923-94.
