# Supplementary material for: Projections of heatwave-attributable mortality under climate change and future population scenarios in China
Source: Lancet Reg Health West Pac. 2022 Sep 5;28:100582. doi: 10.1016/j.lanwpc.2022.100582 (PMC9465423; doi:10.1016/j.lanwpc.2022.100582)
Supplement: Supplementary file 3 [file mmc3.docx]

**Chinese abstract**

**中国气候变化和未来人口情景下热浪归因死亡负担预估**

陈慧琪，赵亮，程亮亮，张雅丽，王会滨，谷魁英，鲍俊哲，杨军，刘钊，黄建斌，陈艺丹，高学杰，徐影，王灿，蔡闻佳，宫鹏，罗勇，梁万年，黄存瑞^*^

**摘要**：

**背景**：在中国，以往对高温相关死亡的大多数预估研究都是利用全球气候模型（GCM）进行模拟研究，这些研究有助于阐明理解气候变化中极端高温事件的健康风险。然而，目前还缺乏使用精细区域气候数据对气候变化相关健康影响的时空变化进行研究。本研究的目标是使用更可信的气候和人口预测来估计不同排放情景下未来热浪导致的死亡人数，并探索这种变化的表象背后的驱动因素。

**方法**：我们从三个CMIP5 GCM模式驱动的区域气候模式中获得气候数据，并在代表性浓度路径（RCP）2.6、RCP4.5和RCP8.5下计算中国未来的热浪。未来的网格化人口数据是基于具有不同生育率的共享社会经济途径（SSP）2假设。通过将气候区特异的暴露反应函数应用于热浪事件期间的死亡人数，我们预测了每种RCP情景下热浪归因死亡人数的规模。并通过分解方法，进一步分析了热浪归因死亡人数变化的驱动因素和主要不确定性来源。此外，我们还比较了1.5°C目标下的归因死亡负担差异，该目标与本世纪中叶实现碳中和密切相关。

**结果**：即使在RCP2.6和RCP4.5情景下，与热浪相关的死亡人数将持续增加至本世纪中叶；而在RCP8.5情景下，归因死亡人数将在21世纪持续增加。在 RCP2.6、RCP4.5、RCP8.5情景下，2090 年将分别有 20,303、35,025、72,260 人死于热浪，并且在三种RCP情景下，与热浪有关的死亡人数的一半都集中在华东和华中地区。从2030年到2060年，气候效应是归因死亡人数增加的主要驱动因素，解释了总变化的78%。此后，RCP8.5情景下，即使人口下降也无法抵消热浪高发和人口老龄化造成的健康损失。尽管1.5°C温升情景下的健康损失比基线期（1986-2005 年）高出 1.6 倍，但与 RCP2.6情景相比，将温升限制在 1.5°C 可以在 2090 年将中国的年死亡负担减少 3,534 人。

**结论**：随着气候变化和人口老龄化的加速，即使在低排放情景下，未来热浪对中国人群的健康危害也可能会持续增加。值得注意的是，如果能够实现乐观的1.5°C温升目标，预计会有显著的健康收益，这表明在本世纪中叶实现碳中和是中国可持续发展的坚定选择。政策制定者需要因地制宜地加强气候减缓政策，同时在技术和基础设施方面增强气候适应能力，尤其是针对脆弱的老年人群。

**基金资助：**国家重点研发计划项目(2018YFA0606200)，威康信托基金(209734/Z/17/Z)，国家自然科学基金重大项目课题（41790471），广东省基础与应用基础研究重大项目（2020B0301030004）

**关键词****：**气候变化，热浪，归因死亡人数，预估，中国
